# Synthesis and Inclusion Properties of a β-Cyclodextrin Heptaphosphoramidate

**DOI:** 10.3390/molecules29122714

**Published:** 2024-06-07

**Authors:** Austin Che, Jayar Espejo, Chang-Chun Ling

**Affiliations:** Department of Chemistry, University of Calgary, Calgary, AB T2N 1N4, Canada; austin.che@ucalgary.ca (A.C.); jayar.espejo@ucalgary.ca (J.E.)

**Keywords:** β-cyclodextrin, phosphoramidate, inclusion complex, steroids

## Abstract

In this study, we report a novel per-6-substituted β-cyclodextrin (**4**) featuring seven phosphoramidate moieties as an innovative host for inclusion. This structurally well-defined host has remarkable water solubility and was isolated in pure form. Analytical techniques such as NMR and ITC were used to probe the molecular interactions with different drug molecules. Our investigations revealed that host **4** can form 2:1 inclusion complexes with various drugs. Further studies showed that the inclusions of drugs by β-CD host (**4**) are mostly enthalpy driven, highlighting the potential roles played by the phosphoramidate functionalities of the host. Comparatively, a per-O2, O3-acetylated analog (**6**) of compound **4** was also obtained, which also shows unusual water solubility but diminished inclusion capability.

## 1. Introduction

**Figure 1:**
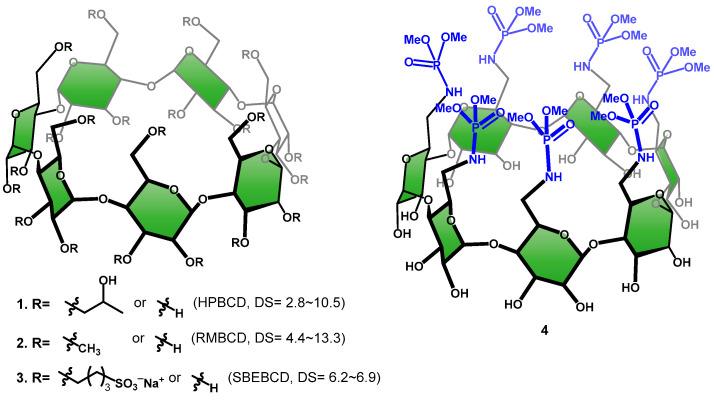
Chemical structures of commercially accessible HPBCD, RMBCD, and SBEBCD, along with a novel structurally well-defined β-CD heptaphosphoramidate (**4**) employed in the current study. The commercially available β-CD products (**1**–**3**) consist of a mixture of analogues with differing degrees of substitution at positions O6, O2, and O3.

Cyclodextrins (CDs) are D-glucose-based macrocycles linked via 1,4-glycosidic linkages, forming α-, β-, and γ-CD with 6, 7, or 8 glucose units, respectively, with β-CD being most commonly used [1,2]. CDs have a frustum shape with primary hydroxyl groups (OH-6’s) on the narrower end (primary face) and secondary hydroxyl groups on the wider end (secondary face). Both faces of CDs feature polar groups, rendering them water-soluble, in their native forms; this makes CDs advantageous over other macrocyclic hosts [3,4,5,6,7,8,9,10]. All native CDs display axial symmetry, with C3-H3 and C5-H5 bonds symmetrically oriented toward the center of the cavity. These covalent bonds, being non-polar, establish a relatively non-polar environment within the molecule. This unique environment allows a CD molecule with the ability to encapsulate organic compounds, such as drugs that contain a significant hydrophobic region, into their cavities when in an aqueous solution. This leads to the formation of host–guest inclusion complexes, which are thermodynamically more favorable [11]. Because of this, CDs have found widespread applications in pharmaceutical industries and others such as foods, agricultural, and textiles industries [12,13]. For instance, CDs, particularly β-CD, have been employed as excipients in formulations to increase the bioavailability of medications. This allows for various improvements such as increased water solubility, air stability, taste masking, and modifying pharmacokinetics [14]. Coupled with its cost-effectiveness, the cavity size of β-CD is advantageous to host hydrophobic fragments in drug molecules, such as isopropyl, *tert*-butyl, cycloalkyl, benzene, etc., rendering β-CD highly appealing to pharmaceutical industries. On the other hand, the water solubility of native β-CD is the lowest of all native CDs. Chemical modifications have been carried out on β-CD in order to improve the inclusion and water solubilities of β-CD derivatives [15]. The three most successful strategies—O-(2-hydroxy)propylation, O-methylation, and O-(4-sulfo)butylation (Figure 1)—yield 2-hydroxypropyl-β-CD [16,17,18] (HPBCD, **1**), randomly substituted methyl β-CD [19,20] (RMBCD, **2**) and sulfobutyl ether β-CD [21,22,23,24] (SBEBCD, **3**,) respectively, which have become the top three major chemically modified β-CDs in the commercial markets worldwide. The CD derivatives offer enhanced water solubility and low toxicity. However, a common drawback of these β-CD derivatives is their commercialization as mixtures with varying degrees of substitution (DS). Studies have shown that the DS of these chemically modified CDs can impact drug inclusion processes, leading to alterations in their complexation capability [25]. For instance, depending on the nature of the guest molecule, the affinity of SBEBCD may vary, exhibiting either an increase or decrease with higher DS [26]. Additionally, as demonstrated by Mennini et al. [27], the presence of substituents interferes with the carrier performance of drug/CD inclusion complexes. In this work, we report the synthesis of a structurally well-defined β-CD heptaphosphoramidate (**4**) as a novel host and its inclusion complexes with selected drugs.

## 2. Results and Discussion

### 2.1. Synthesis and Characterization

Phosphoramidate [28] is an interesting functional group that has found widespread utilities in medicines [29,30,31,32], agriculture [33,34,35,36], and material chemistry [37,38]. However, this chemical functionality has not been introduced to the CD field extensively. We recently described the synthesis of per-6-substituted β-CD heptaphosphoramidate **6** [39]. This involved reacting per-2,3-O-acetyl-6-azido-deoxy-β-CD (**5**) with trialkyl phosphites through a Staudinger-like reaction, resulting in a family of per-2,3-*O*-acetylated β-CD heptaphosphoramidates. For instance, compound **6** was obtained in excellent yields (>86%) by treating compound **5** with trimethyl phosphite, either in dichloromethane at ambient temperature for an extended time, or in toluene at 100 °C for 1 h [39].

**Figure 2:**
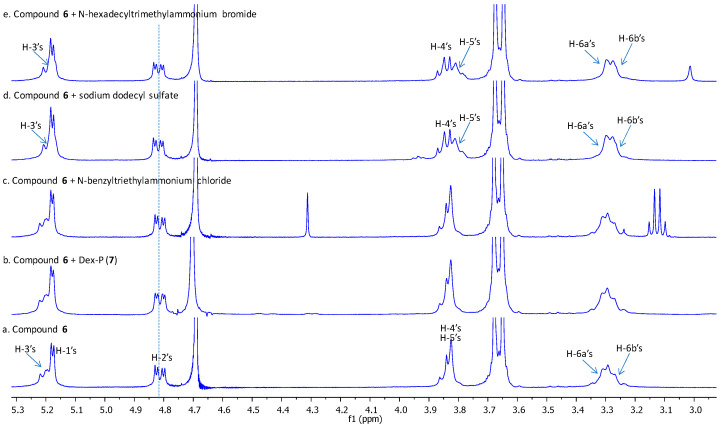
^1^H NMR spectra (2.9–5.3 ppm region) of compounds **6** (spectrum **a**) recorded in D_2_O (400 MHz) and inclusion studies with Dex-P (**7**, spectrum **b**), *N*-benzyltriethylammonium chloride (spectrum **c**), sodium dodecyl sulfate (spectrum **d**), and *N*-hexadecyltriethylammonium bromide (spectrum **e**).

Interestingly, we recently found that despite the presence of 14 *O*-acetyl groups which are relatively hydrophobic, compound **6** is soluble in water. Figure 2 shows the recorded ^1^H NMR spectrum of compound **6** (bottom) in deuterated water. All the H-2 and H-3 protons of glucopyranosyl units were acetylated as they are respectively observed at a highly deshielded region (5.21 ppm for H-3s, and 4.82 ppm for H-2s). The unexpected water solubility of compound **6** could be explained by the presence of extensive hydrogen bond-promoting heteroatoms in the seven phosphoramidate groups.

**Figure 3:**
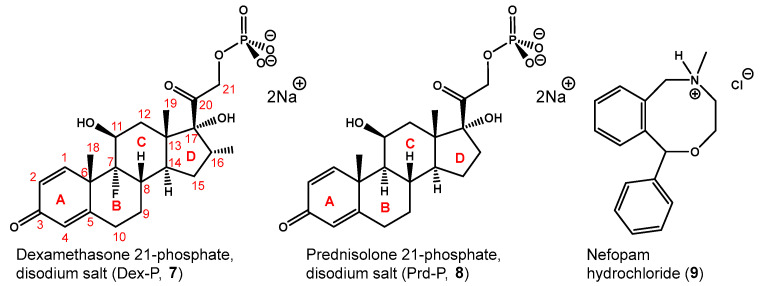
Structures of guest molecules used for inclusion studies in this work: dexamethasone 21-phosphate, disodium salt (Dex-P, **7**), prednisolone 21-phosphate, disodium salt (Prd-P, **8**), and nefopam hydrochloride (**9**).

We tested the ability of compound **6** to form an inclusion complex by ^1^H NMR experiments with disodium dexamethasone 21-phosphate (Dex-P, **7**, Figure 3), and found no evidence of formation of an inclusion complex, as no changes in chemical shifts were observed for any of the protons of the host molecule (See Figure 2, spectrum **b** and **ESI S1**).

Isothermal titration calorimetry (ITC) further confirmed the absence of inclusion of Dex-P (**7**) with compound **6**. Additionally, we also studied the inclusion of compound **6** with other smaller guests, and found no evidence of inclusion complexation formation with *N*-benzyltriethylammonium chloride, but clear evidence of inclusion complex formation with both sodium dodecyl sulfate and *N*-hexadecyltrimethylammonium bromide (See Figure 2, spectrum **c**–**e** and **ESI S1**), based on the chemical shifts of H-3 and H-5 protons inside the cavity of the host as well as H-6a and H-6b protons at the periphery.

**Figure 4:**
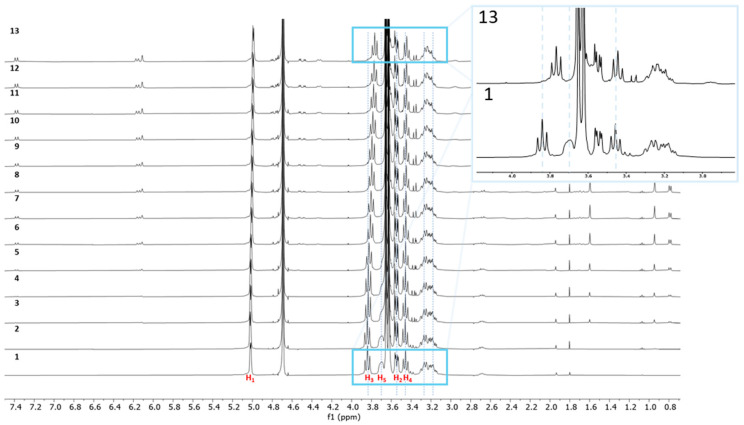
^1^H NMR titration of host **4** (spectrum **1**) with Dex-P (**7**, spectra **2**–**13**) in D_2_O (400 MHz). Both H-3 and H-5 protons of β-CD heptaphosphoramidate (**4**) were observed to shift gradually upfield upon titration with Dex-P (**7**).

**Scheme 1:**
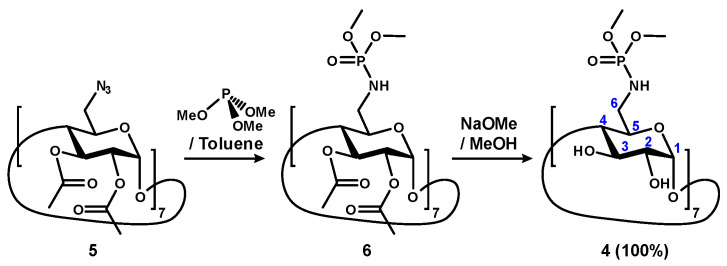
Chemical preparation of per-2,3-*O*-acetylated β-CD heptaphosphoramidate (**6**) and its fully O-deacetylated analogue **4**.

We hypothesized that the presence of 14 *O*-acetate groups at the secondary face might have resulted in certain degrees of self-inclusion, leading to a conformational change of the O-acetate groups that partially block the entrance to the β-CD cavity from the secondary face by large guest molecules such as Dex-P (**7**) and *N*-benzyltriethylammonium chloride; but there are still opportunities to form inclusion complexes with more linear guest molecules such as sodium dodecyl sulfate and *N*-hexadecyltrimethylammonium bromide. We therefore removed all the 14 O-acetate groups via a Zemplén transesterification in anhydrous methanol in the presence of sodium methoxide (Scheme 1). The corresponding compound **4** was obtained, purified via size exclusion column chromatography, followed by lyophilization, and isolated in a quantitative yield. Characterization of compound **4** was first conducted using ^1^H NMR, assisted by 2D ^1^H-^1^H COSY. In the ^1^H NMR spectrum, sharp and uniform peaks were observed (Figure 4, bottom spectrum and ESI **S2**). Only one set of proton signals for the glucopyranose units were observed, confirming the symmetrical nature of compound **4**. The extracted coupling constants were *J*_1,2_ = 3.8 Hz, *J*_2,3_ = 10.0 Hz, *J*_3,4_ = 9.3 Hz, *J*_4,5_ = 9.3 Hz, consistent with the expected ^4^*C*_1_ chair conformation. Additionally, both H-2 and H-3 protons were observed upfield at 3.56 and 3.85 ppm, respectively, affirming the clean removal of all the electron-withdrawing *O*-acetyl groups. Complex multiplets for H-6_a_ and H-6_b_ were also observed, attributed to the direct attachment of the methyl phosphoramidate group and the additional heteronuclear coupling with the phosphorus moiety. Furthermore, two distinct singlet peaks for the methyl groups were observed at 3.67 and 3.63 ppm. Analysis of the ^13^C NMR (See **ESI S3**), supported by the ^1^H-^13^C HSQC spectrum (See **ESI S5**), showed eight peaks corresponding to the six glucopyranose carbons and two distinct types of methyl groups linked to the phosphoramidate, attributed to restricted rotation. Consistent with previous findings, the C-5s also exhibited long-range coupling with the phosphorus moiety, appearing as a doublet (71.2 ppm, *J* = 6.3 Hz), whereas C-6 did not couple despite direct attachment [39]. Both methyl phosphoramidate groups displayed coupling to the phosphorus, appearing as a pair of doublets at 53.54 and 53.48 ppm (*J* = 6.1 and 6.2 Hz). ^31^P NMR also shows a single oxidized phosphorus species at 14.4 ppm confirming the presence of seven identical phosphorus groups (see **ESI**, **S6**). The final confirmation of the identity of compound **4** was supported by electrospray high-resolution mass spectrometry (positive, (see **ESI**, **S23**)) which revealed a peak at *m*/*z* 1906.4540, matching closely to the calculated sodium adduct of expected compound **4**, with a molecular formula of C_56_H_112_N_7_O_49_P_7_ (*m*/*z* 1906.4543, M + Na^+^).

### 2.2. Inclusion Studies

With pure compound **4** in hand, we next tested its ability to form an inclusion complex with Dex-P (**7**) using ^1^H NMR titrations. As illustrated in Figure 4, upon titration of Dex-P (**7**) into a solution of β-CD heptaphosphoramidate (**4**) in deuterated water, the H-3 and H-5 protons of all glucopyranosyl units exhibited a progressively upfield shift, while other protons, such as H-2, H-4, and H-6_a_/H-6_b_, showed minimal changes. This strongly suggests the formation of an inclusion complex between compound **4** and Dex-P (**7**) in water, as H-3s and H-5s are the most shifted protons, located within the interior of the β-CD cavity; these changes in the observed chemical shifts indicate an interaction with Dex-P (**7**).

**Figure 5:**
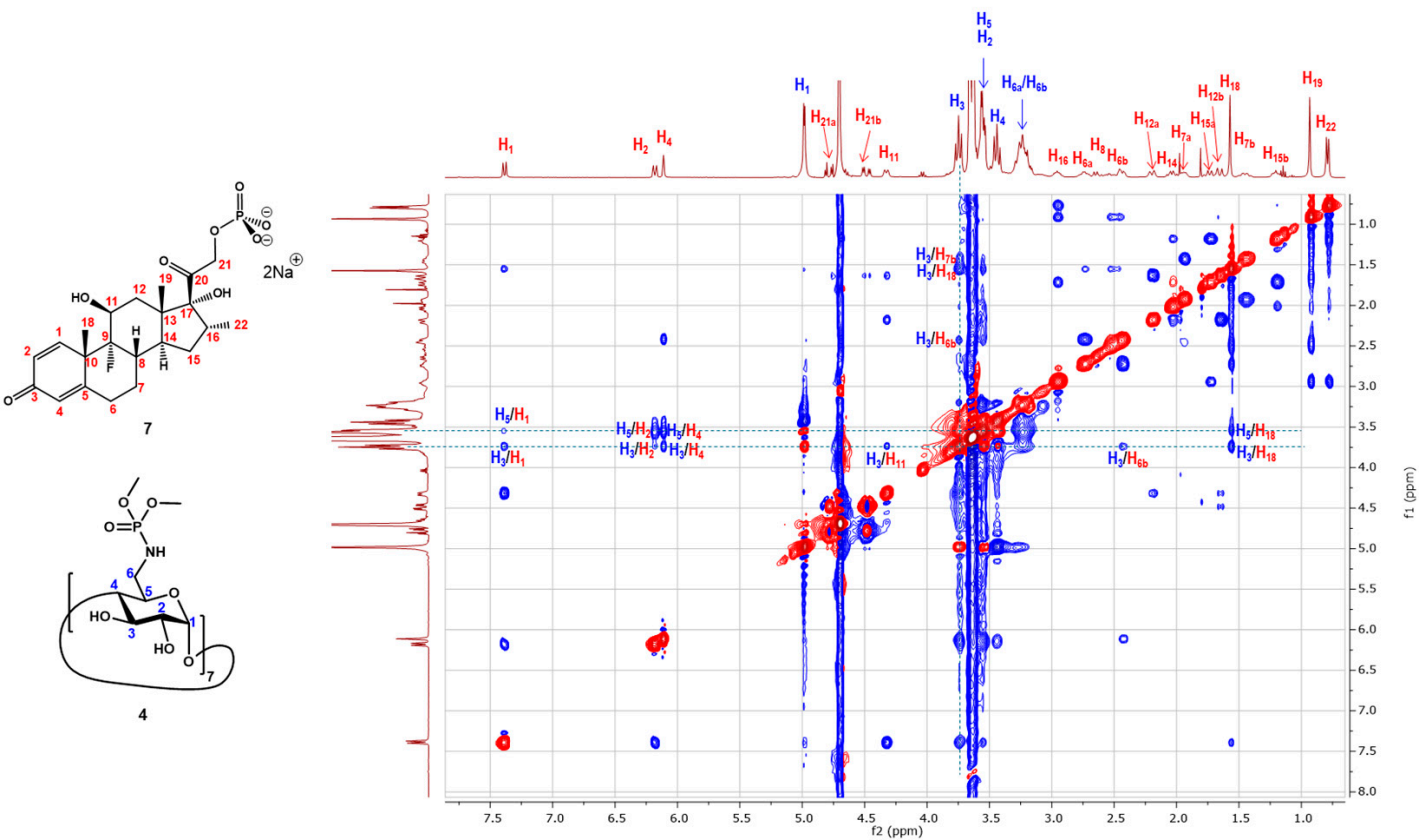

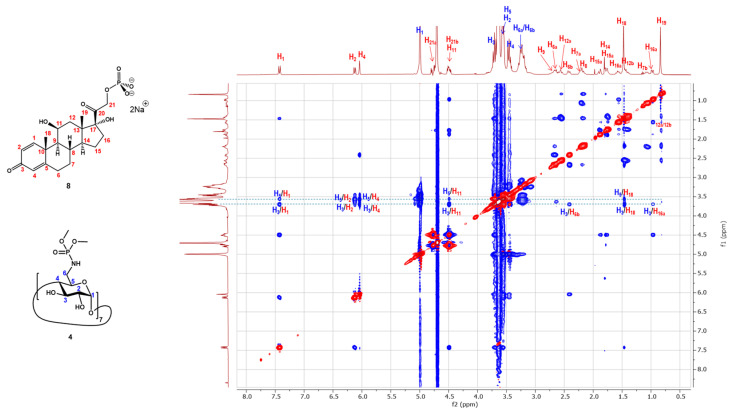
2D ^1^H-^1^H ROESY NMR experiments of host **4** with Dex-P (**7**, **top** spectrum) and Prd-P (**8**, **bottom** spectrum) in D_2_O (400 MHz). The correlations in the highlighted boxes indicate ROE signals between the H-3 and H-5 protons of β-CD heptaphosphoramidate (**4**) and Dex-P (**7**) and Prd-P (**8**).

To confirm the inclusion between β-CD heptaphosphoramidate (**4**) and Dex-P (**7**), we conducted a 2D ^1^H-^1^H ROESY experiment (Figure 5). The experiment revealed extensive ROE correlations (highlighted boxes) between the H-3 and H-5 protons of the host (**4**) and the protons of Dex-P (**7**) at 7.38, 6.18, 6.11, and 4.32 ppm, along with several others in the 0.75–3.1 ppm region, corresponding to the four rings (A–D) of the Dex-P molecule. However, no evidence was found to indicate ROE between the H-6_a_ and H-6_b_ protons of the glucopyranosyl units with the protons of Dex-P (**7**), nor between the methoxy groups of the phosphoramidate groups and protons of Dex-P (**7**). This suggests that the Dex-P (**7**) guest molecule might predominantly reside near the lower part of the cavity of β-CD heptaphosphoramidate (**4**). This can be explained by the well-known fact that β-CD hosts typically form a 2:1 inclusion complex with a steroid molecule, as demonstrated by the crystal structure between cholesterol and native β-CD [40] and another study [41].

We hypothesize that Dex-P (**7**) would primarily interact with the lower part of the cavity of β-CD heptaphosphoramidate (**4**), as Dex-P (**7**) has a shorter length compared to cholesterol due to the truncation of the side-chain; thus, it does not have the dimension to reach the cavity space of the two opposing CDs where the C-6s and phosphoramidates are situated.

**Figure 6:**
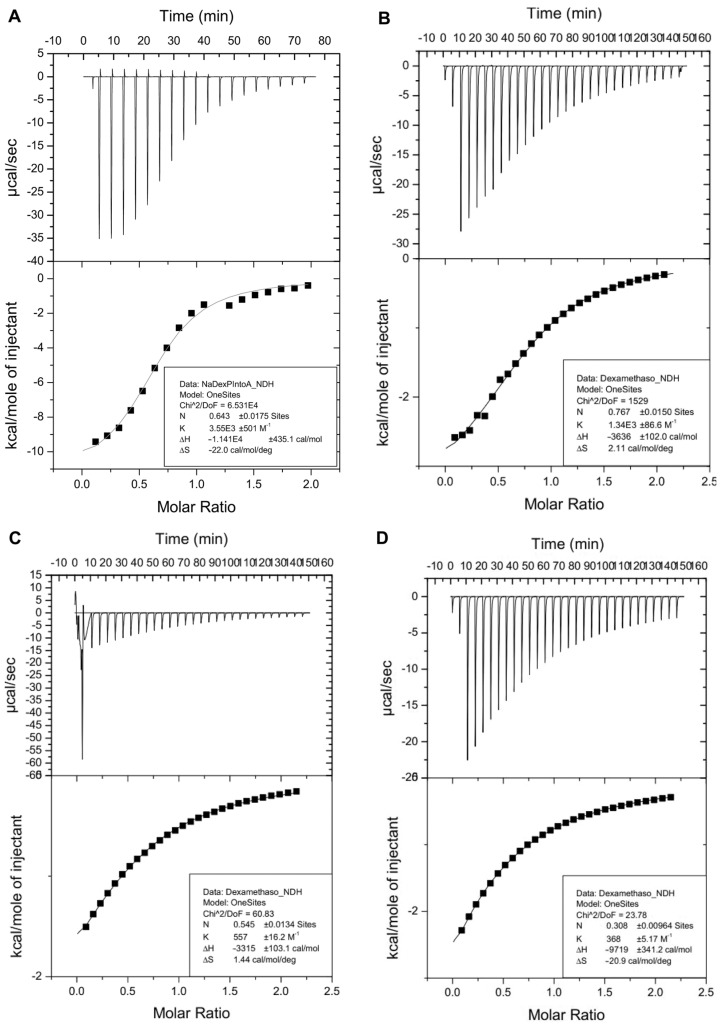
Isothermal calorimetry thermograms of titrating a solution of Dex-P (**7**, 30 mM) into (**A**) β-CD heptaphosphoramidate (**4**, 3.0 mM), (**B**) HPBCD (**1**, DS = 2.8~10.5, 3.0 mM), (**C**) RMBCD (**2**, DS = 4.4~13.3, 3.0 mM), (**D**) SBEBCD (**3**, DS = 6.2~6.9, 3.0 mM). All titrations were carried out in a potassium phosphate buffer (pH 7.4) and a 2.0 mL injection volume. Integrated data also show the calculated binding constant (K_a_), enthalpy (ΔH), and entropy (ΔS).

Isothermal titration calorimetry (ITC) was also used to characterize the inclusion of Dex-P (**7**) by β-CD heptaphosphoramidate (**4**). The titration was carried out by adding aliquots (2.0 μL) of a stock solution of Dex-P (**7**, 30.0 mM) in a phosphate buffer solution (pH~7.4) to a solution of β-CD heptaphosphoramidate (**4**, 3.0 mM) in the same buffer. The thermograms of the titration experiment are shown in Figure 6A. As depicted, the binding of Dex-P (**7**) to β-CD heptaphosphoramidate (**4**) is exothermic, confirming the productive binding interaction between the host and guest. The determined thermodynamic parameters based on the fitted data correlate well with the formation of a 2:1 complex, as the calculated N value is close to 0.5 (N = 0.6, Figure 6A), with a binding association constant (K_a_) of 3.6 × 10^3^ M^−1^. Additionally, an enthalpy change (ΔH) of −11.4 kcal/mol and an entropy loss (ΔS) of −22.0 cal/(mol·K) were determined for the binding. Based on these data, the calculated Gibbs energy change was −4.8 kcal/mol (Table 1), which confirms that the formation of an inclusion complex is strongly favorable (spontaneous). 

To further compare the complex of Dex-P (**7**) with the β-CD heptaphosphoramidate (**4**), we carried out other ITC experiments of Dex-P (**7**) with commercial HPBCD (**1**, DS = 2.8~10.5), RMBCD (**2**, DS = 4.4~13.3), and SBEBCD (**3**, DS = 6.2~6.9). As illustrated in Figure 6B–D and Table 1, despite being mixtures containing different degrees of substitutions, all three commercial hosts exhibit binding to Dex-P (**7**). However, they demonstrate weaker binding association constants (apparent K_a_) compared to β-CD heptaphosphoramidate (**4**, K_a_ = 3.6 × 10^3^ M^−1^), with HPBCD (**1**) being the second best (K_a_ = 1.3 × 10^3^ M^−1^). Both RMBCD (**3**) and SBEBCD (**3**) bind Dex-P (**7**) approximately one order of magnitude weaker (K_a_ = 5.6 × 10^2^ M^−1^ for RMBCD (**3**), and K_a_ = 3.6 × 10^2^ M^−1^ for SBEBCD (**3**)). It should be noted that the Dex-P (**7**):β-CD heptaphosphoramidate (**4**) system exhibited the most exothermic enthalpy change and most entropy loss during the inclusion complex formation (Table 1). This suggests that the inclusion of Dex-P (**7**) to β-CD heptaphosphoramidate (**4**) is mainly enthalpy driven. We hypothesize that the presence of seven phosphoramidate groups at the primary face of β-CD cavity might be contributing to this difference, as the drug could dynamically change conformations to engage in productive hydrogen bonding with the phosphate group of included Dex-P (**7**).

**Figure 7:**
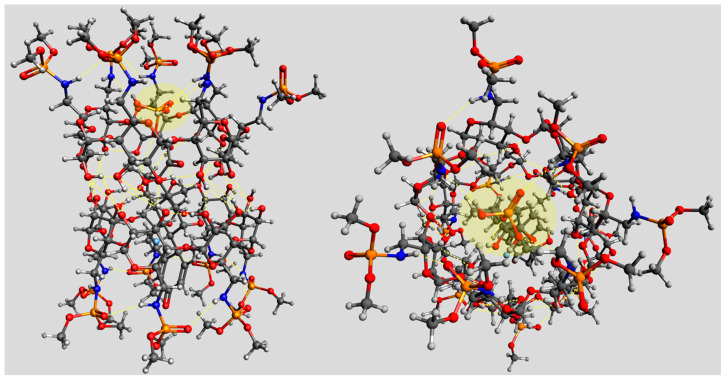
A molecular model of two opposing β-CD heptaphosphoramidate host (**4**) with a docked Dex-P (**7**) in protonated forms. (**Left**): side view; (**right**): focused top–bottom view. Yellow discontinued lines indicate H-bonds. Potential H-bonds between the phosphate group of Dex-P (**7**) and a 6-*N*-phosphoramidate group can be established as indicated by the discontinued yellow lines in the highlighted oval area. Color codes of atom types: dark gray (carbon), light gray (hydrogen), red (oxygen), blue (nitrogen), orange (phosphorus) and cyan (fluoride).

Figure 7 shows a molecular model of the formed 2:1 complex between two β-CD heptaphosphoramidate hosts (**4**) and a docked Dex-P (**7**) molecule. The two opposing β-CD hosts can interact with each other via a complex network of intra/intermolecular hydrogen bonds. This creates an elongated groove for the productive binding of Dex-P (**7**) to take place. The hydrophobic groups of Dex-P guest (**7**) can effectively interact with the two cavities of β-CD hosts via non-covalent interactions. The phosphate group of Dex-P (**7**) can indeed be extended to the region near the primary face of one β-CD host to allow dynamic hydrogen bonds to take place with the N-H group of a 6-*N*-phosphoramidate (Figure 7). We also noticed that the phosphate group of Dex-P (**7**) and the nearby 6-*N*-phosphoramidates could also form an O/N-enriched environment for a possible chelation of sodium cation. Thus, in addition to the putative hydrogen bonding, cation-mediated chelations could also be used to account for the strong enthalpy-driven inclusion between guest and host.

To broaden our understanding of the inclusion capacity of the β-CD heptaphosphoramidate host (**4**), we then investigated its interaction with disodium prednisolone-21-phosphate (**8**, Prd-P) and nefopam hydrochloride (**9**) through both 2D ^1^H-^1^H ROESY NMR experiment and ITC. Dex-P (**7**) and Prd-P (**8**) are analogs of each other, and the main differences reside at C-7 and C-16, with Dex-P (**7**) having a fluoride substitution at C-7 and methyl substitution at C-16. From the recorded 2D ^1^H-^1^H ROESY NMR spectrum, we noticed comparable correlation peaks between protons across the four rings of Prd-P (**8**) and the H-3s and H-5s of the β-CD heptaphosphoramidate host (**4**), indicating that Prd-P (**8**) binds to the host (**4**) in a similar manner to Dex-P (**7**)**.** The determined thermodynamic parameters from ITC (Table 2, See **ESI S17**) also revealed the binding process is enthalpy driven (ΔH° = −11.2 kcal/mol, ΔS° = −23.5 cal/(mol·K)). However, the determined binding association constant (K_a_ = 1.2 × 10^3^ M^−1^) is slightly weaker than that between Dex-P (**7**) and host **4**. This aligns with the lower hydrophobicity of Prd-P (**8**) in comparison to Dex-P (**7**), suggesting that the less hydrophobic nature of Prd-P (**8**) would result in poorer binding. Conversely, ITC demonstrated that Prd-P (**8**) binds to SBEBCD (**3**) with a comparable affinity to host **4** (See **ESI S19**), but exhibits weaker affinity towards HPBCD (**1**) compared to host (**4**) (See **ESI S18**).

**Figure 8:**
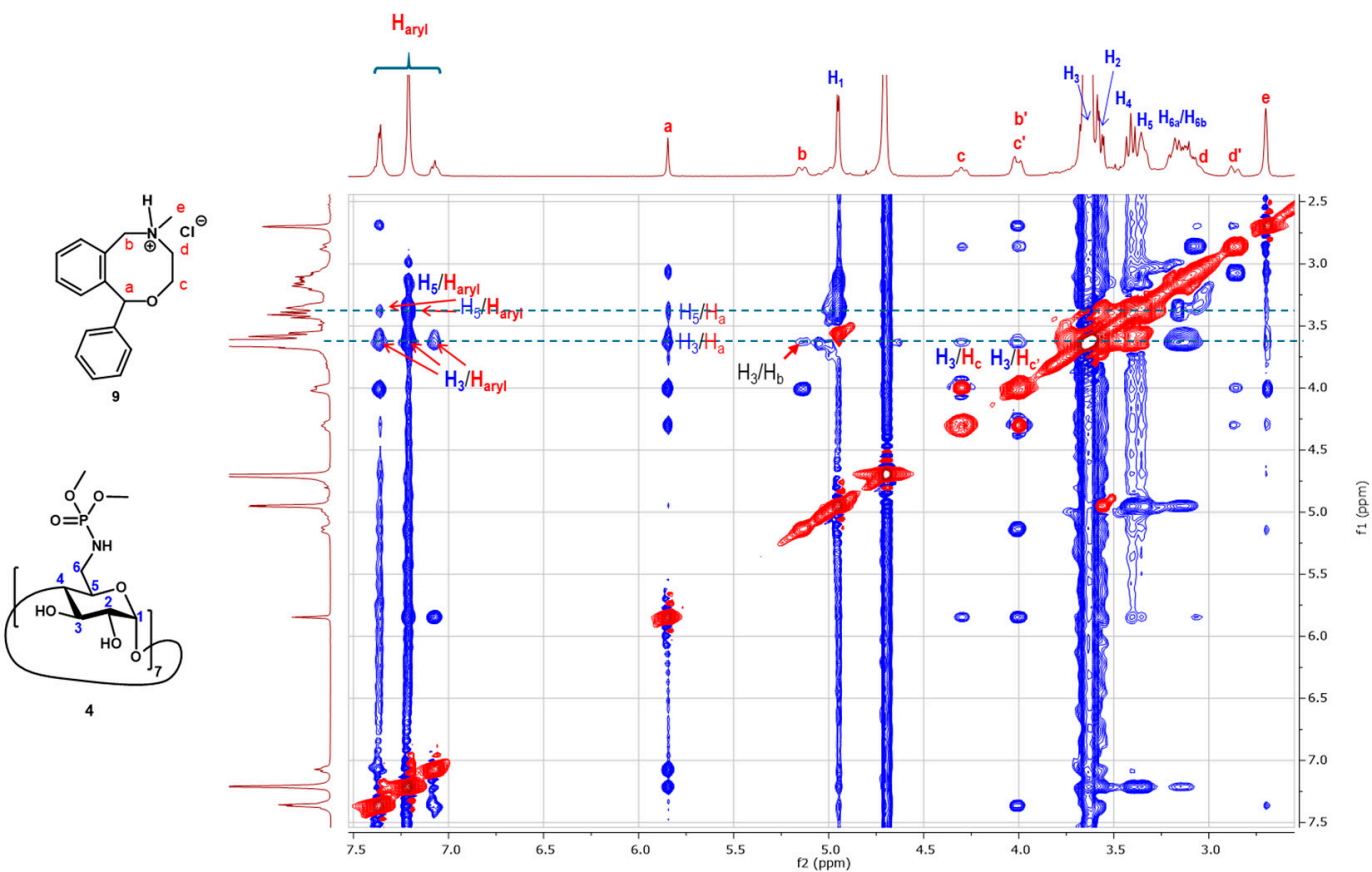
2D ^1^H-^1^H ROESY NMR experiments of host **4** with nefopam hydrochloride (**9**) in D_2_O (400 MHz). The highlighted boxes indicate ROE signals between the H-3 and H-5 protons of β-CD heptaphosphoramidate (**4**) with nefopam hydrochloride (**9**).

The recorded 2D ^1^H-^1^H ROESY NMR spectrum (Figure 8) between nefopam hydrochloride and host (**4**) also showed extensive ROE correlations between the H-3 and H-5 protons of the host with the aromatic protons (7.07–7.38 ppm), non-aromatic protons (3.02–5.86 ppm), and the *N*-methyl group (2.72 ppm) which confirms the formation of an inclusion complex. An ITC experiment was also carried out to determine the thermodynamic parameters (See **ESI S20**). As shown in Table 3, the binding process between host **4** and nefopam hydrochloride is also strongly enthalpy driven (ΔH° = −13.0 kcal/mol, ΔS° = −26.9 cal/(mol·K)). The determined binding association constant (K_a_) is 4.6 × 10^3^ M^−1^ which is stronger than the inclusion complexes formed between nefopam hydrochloride and HPBCD (**1**, K_a_ = 1.6 × 10^3^ M^−1^, see **ESI S21**) and SBEBCD (**3**, K_a_ = 1.2 × 10^3^ M^−1^ see **ESI S22**).

## 3. Conclusions

We have reported a novel β-CD host (**4**) and its per-O2,O3-acetylated analog (**6**) that are persubstituted at the primary face with *N*-phosphoramidate functionalities. These novel hosts can be easily synthesized and obtained in pure form. The per-O2,O3-deacetylated host (**4**) showed increased water solubility compared to native β-CD, presenting an advantage. It also demonstrated an excellent ability to form inclusion complexes with different drug molecules. On the other hand, although the per-O2,O3-acetylated analog (**6**) showed a remarkable and unexpected water solubility, it has much reduced inclusion capability, likely as a result of partial blockage to the hydrophobic cavity by the *O*-acetates. Compared to other commercially available β-CD hosts, the use of novel host **4** could show many advantages because of its well-defined structure. For example, when used in different drug formulations, their compositions, physico-chemical properties, toxicity, pharmacokinetics, and others can be well characterized and reproducible. Overall, our findings shed light on the promising potential of β-CD host **4** as a versatile platform for drug delivery, owing to its well-defined structure, aqueous solubility, and enthalpy-driven inclusion behavior facilitated by the incorporated phosphoramidate motifs.

## 4. Materials and Methods

### 4.1. Chemical Synthesis

All commercial reagents were used as supplied unless otherwise stated. Analytical thin layer chromatography was performed on Silica Gel 60-F_254_ (Sigma-Aldrich^®^ TLC Plates, Sigma-Aldrich, St. Louis, MO, USA) with detection by quenching of fluorescence and/or by charring with 5% sulfuric acid in water or with a ceric ammonium molybdate dip. Column chromatography was performed on Silica Gel 60 (Silicycle, ON, Canada). Organic solutions from extractions were concentrated under vacuum with the assistance of a heat bath. ^1^H NMR spectra were recorded at 400 MHz and ^13^C NMR spectra were recorded at 100 MHz on a Bruker spectrometer. Chemical shifts δ_H_ and δ_C_ are reported in δ (ppm) and referenced to residual HDO (*δ*_H_ 4.79) of the D_2_O solvent and external acetone (*δ*_C_ 29.9). First-order coupling constants were reported in Hz for proton nuclei. ^1^H and ^13^C NMR spectra were assigned with the assistance of DEPTQ, COSY, and HSQC spectra. High-resolution ESI-QTOF mass spectra were recorded on an Agilent 6520 Accurate Mass Quadrupole Time-of-Flight LC/MS spectrometer.

**Compound 4**

Compound **6** (0.30 g, 0.12 mmol) was dissolved in anhydrous methanol (3.0 mL). A solution of 1.5 M sodium methoxide was added until the pH ~9 and was stirred overnight. The compound was purified by column chromatography on Sephadex LH20 using methanol–water (1:1) before being subjected to cryodesiccation to obtain compound **4** as a white solid (0.23 g, 100%). [α]_25_^D^ + 53.4 (*c* 0.51, CH_3_OH). ^1^H NMR (400 MHz, D_2_O) δ 5.03 (d, *J* = 3.8 Hz, 7H, 7 × H-1), 3.85 (dd, *J* = 10.0, 8.9 Hz, 7H, 7 × H-3), 3.70 (br ddd, 7H, 7 × H-5), 3.67 (s, 3H, 7H, 7 × OCH_3_), 3.63 (s, 7H, 7 × OCH_3_), 3.56 (dd, *J* = 10.0, 3.6 Hz, 7H, 7 × H-2), 3.47 (dd, *J* = 9.3, 9.3 Hz, 7H, 7 × H-4), 3.34–3.13 (m, 14H, 7 × H-6_a_, 7 × H-6_b_). ^13^C NMR (101 MHz, D_2_O) δ 101.4, 81.6, 72.7, 71.9, 71.3, 71.2, 53.54, 53.48, 41.0. ^31^P NMR (162 MHz, D_2_O) δ 14.44. HRMS (ESI-QTOF, positive) *m*/*z* calc’d for C_56_H_112_N_7_O_49_P_7_ (M + Na^+^): 1906.4543; found 1906.4538.

### 4.2. Isothermal Calorimetry 

The binding between guests Dex-P, Nef-HCl, Roc-Br, Pred-P and hosts **2**, βCD, SBE-βCD, and RM-βCD was investigated through ITC experiments carried out with a Nano ITC2G calorimeter (TA Instruments, New Castle, DE, USA) at a constant temperature of 25 °C. Prior to the titration experiments, both solutions were degassed under a vacuum for 20 min. The experiment consisted typically of injecting 2 µL of guest ligand (30.0 mM) into the calorimetric cell that contains the β-CD solution (3.0 mM) by using a computer-controlled Hamilton micro-syringe. The reference cell was loaded with potassium phosphate buffer (pH 7.4). The interval of 300 s between injections was set to allow the heat signal to return to the baseline. During the titration, the reaction mixture was continuously stirred at 500 rpm to ensure proper mixing after each injection. The integrated heat data, after the correction for control, were analyzed using Origin software (version 7.0). The binding constant (K), the binding stoichiometry (n), the change in enthalpy (∆H), and the change in entropy (∆S) were thus obtained.

### 4.3. Molecular Model

The three-dimensional coordinates of the native ß-CD scaffold were taken from its crystal structure [42] and modified using Accelrys DS Viewer Pro (Version 6.0) to obtain the molecular model of ß-CD heptaphosphoramidate **4**. The molecular model of Dex-P (**7**) was also constructed using Accelrys DS Viewer Pro. The dimer model of ß-CD host (**4**) was constructed in Accelrys DS Viewer Pro according to the crystal structure of reported cholesterol/ß-CD complex [40] and Dex-P (**7**) model was docked into the formed dimer. The formed inclusion complex was then exported to Avogadro (Version 1.20) [43,44] for initial energy minimization using the built-in UFF forcefield. The coordinates of the resulting complex were then exported to Gaussian (Version 09w) [45] and minimized by using the semi-empirical PM3 method.

## Figures and Tables

**Table 1:** Thermodynamic parameters of Dex-P with CD host **4** and other commercial CD hosts HPBCD, SBEBCD, and RMBCD.

	4	HPBCD	SBEBCD	RMBCD
K_a_ (M^−1^)	3.6 × 10^3^	1.3 × 10^3^	3.7 × 10^2^	5.6 × 10^2^
ΔH° (kcal/mol)	−11.4	−3.6	−9.7	−3.3
ΔS° (cal/(mol·K))	−22.0	2.1	−20.9	1.4
ΔG° (kcal/mol)	−4.8	−4.3	−3.5	−3.8

**Table 2:** Thermodynamic parameters of Prd-P (**8**) with CD host **4** and other commercial CD hosts HPBCD and SBEBCD.

	4	HPBCD	SBEBCD
K_a_ (M^−1^)	1.2 × 10^3^	4.0 × 10^3^	1.9 × 10^3^
ΔH° (kcal/mol)	−11.2	−1.7	−1.7
ΔS° (cal/mol·K)	−23.5	10.8	9.2
ΔG° (kcal/mol)	−4.2	−4.9	−4.5

**Table 3:** Thermodynamic parameters of nefopam hydrochloride with CD host **4** and other commercial CD hosts HPBCD and SBEBCD.

	4	HPBCD	SBEBCD
K_a_ (M^−1^)	4.6 × 10^3^	1.6 × 10^3^	1.2 × 10^3^
ΔH° (kcal/mol)	−13.0	−3.4	−3.4
ΔS° (cal/(mol·K))	−26.9	3.4	7.2
ΔG° (kcal/mol)	−5.0	−4.4	−5.6

## Data Availability

Data are contained in the manuscript and Supplementary Materials.

## Supplementary Materials

The following supporting information can be downloaded at: https://www.mdpi.com/article/10.3390/molecules29122714/s1. Figure S1: ^1^H NMR Spectra of compound **6** (spectrum **a**) and inclusion studies with Dex-P (**7**, spectrum **b**), *N*-benzyltriethylammonium chloride (spectrum **c**), sodium dodecyl sulfate (spectrum **d**) and *N*-hexadecyltrimethylammonium bromide (spectrum **e**) in D_2_O; Figure S2: ^1^H NMR spectrum of compound **4** in D_2_O; Figure S3: ^13^C NMR spectrum of compound **4** in D_2_O; Figure S4: ^1^H-^1^H COSY NMR spectrum of compound **4** in D_2_O; Figure S5: ^1^H-^13^C HSQC NMR spectrum of compound **4** in D_2_O; Figure S6: ^31^P-NMR spectrum of compound **4** in D_2_O; Figure S7: ^1^H NMR spectrum of compound **4** + dexamethasone sodium phosphate (Dex-P, 7) in D_2_O; Figure S8: ^1^H–^1^H ROESY spectrum of compound **4** + dexamethasone sodium phosphate (DEX-P, **7**) in D_2_O; Figure S9: ^1^H NMR spectrum of compound **4** + nefopam HCl (**9**) in D_2_O; Figure S10: ^1^H–^1^H ROESY spectrum of compound **4** + nefopam HCl (**9**) in D_2_O; Figure S11: ^1^H NMR spectrum of compound **4** + prednisolone disodium phosphate (Prd-P, **8**) in D_2_O; Figure S12: ^1^H–^1^H ROESY spectrum of compound **4** + prednisolone disodium phosphate (Prd-P, **8**) in D_2_O; Figure S13: ITC titration of Dex-P (**7**) into compound **4**; Figure S14: ITC titration of Dex-P (**7**) into HPBCD (**1**); Figure S15: ITC titration of Dex-P (**7**) into RMBCD (**2**); Figure S16: ITC titration of Dex-P (**7**) into SBEBCD (**3**); Figure S17: ITC titration of Prd-P (**8**) into compound **4**; Figure S18: ITC titration of Prd-P (**8**) into HPBCD (**1**); Figure S19: ITC titration of Prd-P (**8**) into SBEBCD (**3**); Figure S20: ITC titration of nefopam HCl (**9**) into compound **4**; Figure S21: ITC titration of nefopam HCl (**9**) into HPBCD (**1**); Figure S22: ITC titration of nefopam HCl (**9**) into SBEBCD (**3**); Figure S23: HRMS Spectra of Compound **4**.
