# Supplementary material for: Synthesis and Inclusion Properties of a β-Cyclodextrin Heptaphosphoramidate
Source: Molecules. 2024 Jun 7;29(12):2714. doi: 10.3390/molecules29122714 (PMC11205585; doi:10.3390/molecules29122714)
Supplement: Supplementary file 1 [file molecules-29-02714-s001.zip › molecules-3013033-supplementary.pdf]

Supporting Information

Synthesis and Inclusion Properties of a β-Cyclodextrin Heptaphosphoramidate

Austin Che,[†] Jayar Espejo[†], and Chang-Chun Ling[*]

Department of Chemistry, University of Calgary, Calgary, Alberta T2N 1N4, Canada

E-mail: *ccling@ucalgary.ca*

Contents

I. NMR Spectra 4

NMR Spectra of compound **6** 4

Figure S1: ^{1}H NMR Spectra of compound **6** (spectrum **a**) and inclusion studies with Dex-P (**7**, spectrum **b**), *N*-benzyltriethylammonium chloride (spectrum **c**), sodium dodecyl sulfate (spectrum **d**) and *N*-hexadecyltrimethylammonium bromide (spectrum **e**) in D_2O 4

NMR Spectra of compound **4** 5

Figure S2: ^{1}H NMR Spectrum of compound **4** in D2O 5

Figure S3: ^{13}C NMR Spectrum of compound **4** in D_2O 6

Figure S4: ^{1}H-^{1}H COSY NMR Spectrum of compound **4** in D_2O 7

Figure S5: ^{1}H-^{13}C HSQC NMR Spectrum of compound **4** in D_2O 8

Figure S6: ^{31}P-NMR Spectrum of compound **4** in D_2O 9

Figure S7: ^{1}H NMR Spectrum of compound **4** + Dexamethasone Sodium Phosphate (Dex-P, **7**) in D_2O 10

Figure S8: ^{1}H–^{1}H ROESY Spectrum of compound **4** + Dexamethasone Sodium Phosphate (DEX-P, **7**) in D_2O 11

Figure S9: ^{1}H NMR Spectrum of compound **4** + Nefopam HCl (**9**) in D_2O 12

Figure S10: ^{1}H–^{1}H ROESY Spectrum of compound **4** + Nefopam HCl (**9**) in D_2O 13

Figure S11: ^{1}H NMR Spectrum of compound **4** + Prednisolone Disodium Phosphate (Prd-P, **8**) in D_2O 14

Figure S12: ^{1}H–^{1}H ROESY Spectrum of compound **4** + Prednisolone Disodium Phosphate (**8**) in D_2O 15

II. Isothermal Titration Calorimetry (ITC) 16

Figure S13: ITC Titration of Dex-P (**7**) into compound **4.** 16

Figure S14: ITC Titration of Dex-P (**7**) into HPBCD (**1**). 17

Figure S15: ITC Titration of Dex-P (**7**) into RMBCD (**2**). 18

Figure S16: ITC Titration of Dex-P (**7**) into SBEBCD (**3**). 19

Figure S17: ITC Titration of Prd-P (**8**) into compound **4**. 20

Figure S18: ITC Titration of Prd-P (**8**) into HPBCD (**1**). 21

Figure S19: ITC Titration of Prd-P (**8**) into SBEBCD (**3**). 22
Figure S20: ITC Titration of Nefopam HCl (**9**) into compound **4** 23
Figure S21: ITC Titration of Nefopam HCl (**9**) into HPBCD (**1**). 24
Figure S22: ITC Titration of Nefopam HCl (**9**) into SBEBCD (**3**). 25
HRMS Spectra 26
Figure S23: HRMS Spectra of Compound **4**........ 26

I. NMR Spectra

NMR Spectra of compound **6**

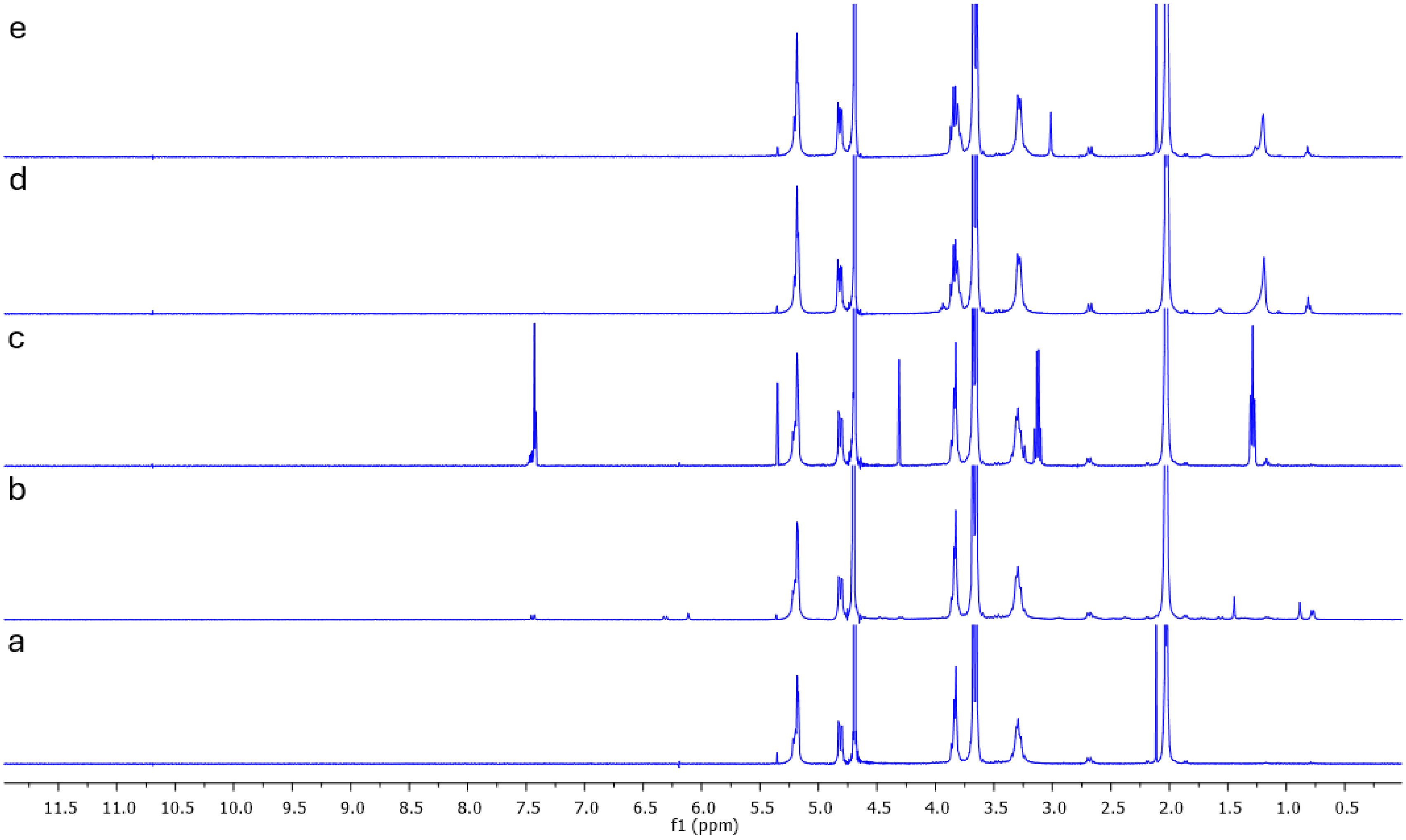

Figure S1: ^{1}H NMR Spectra of compound **6** (spectrum **a**) and inclusion studies with Dex-P (**7**, spectrum **b**), *N*-benzyltriethylammonium chloride (spectrum **c**), sodium dodecyl sulfate (spectrum **d**) and *N*-hexadecyltrimethylammonium bromide (spectrum **e**) in D_2O.

NMR Spectra of compound **4**

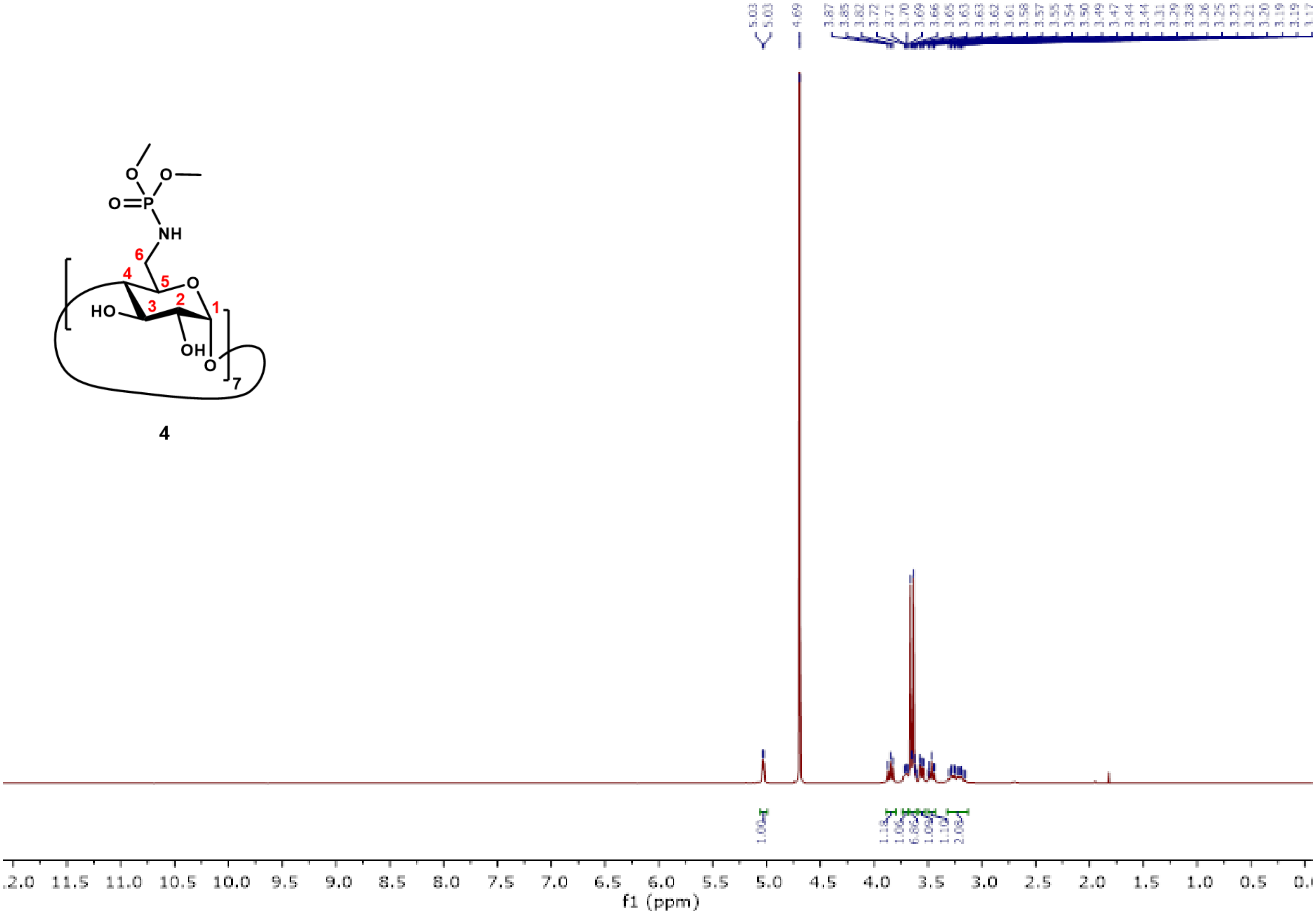

Figure S2: ^{1}H NMR Spectrum of compound **4** in D_2O.

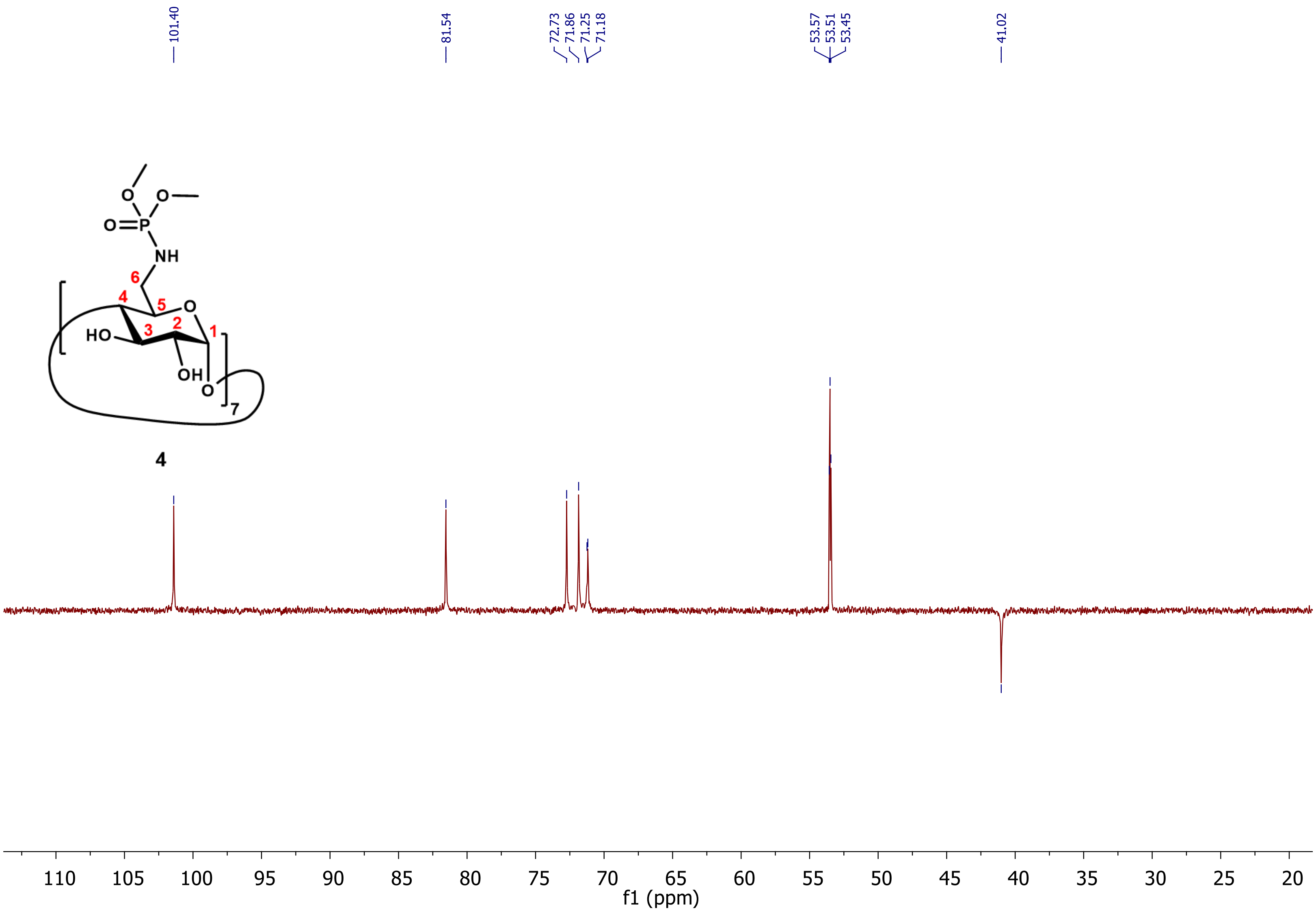

Figure S3: ^{13}C NMR Spectrum of compound **4** in D_2O.

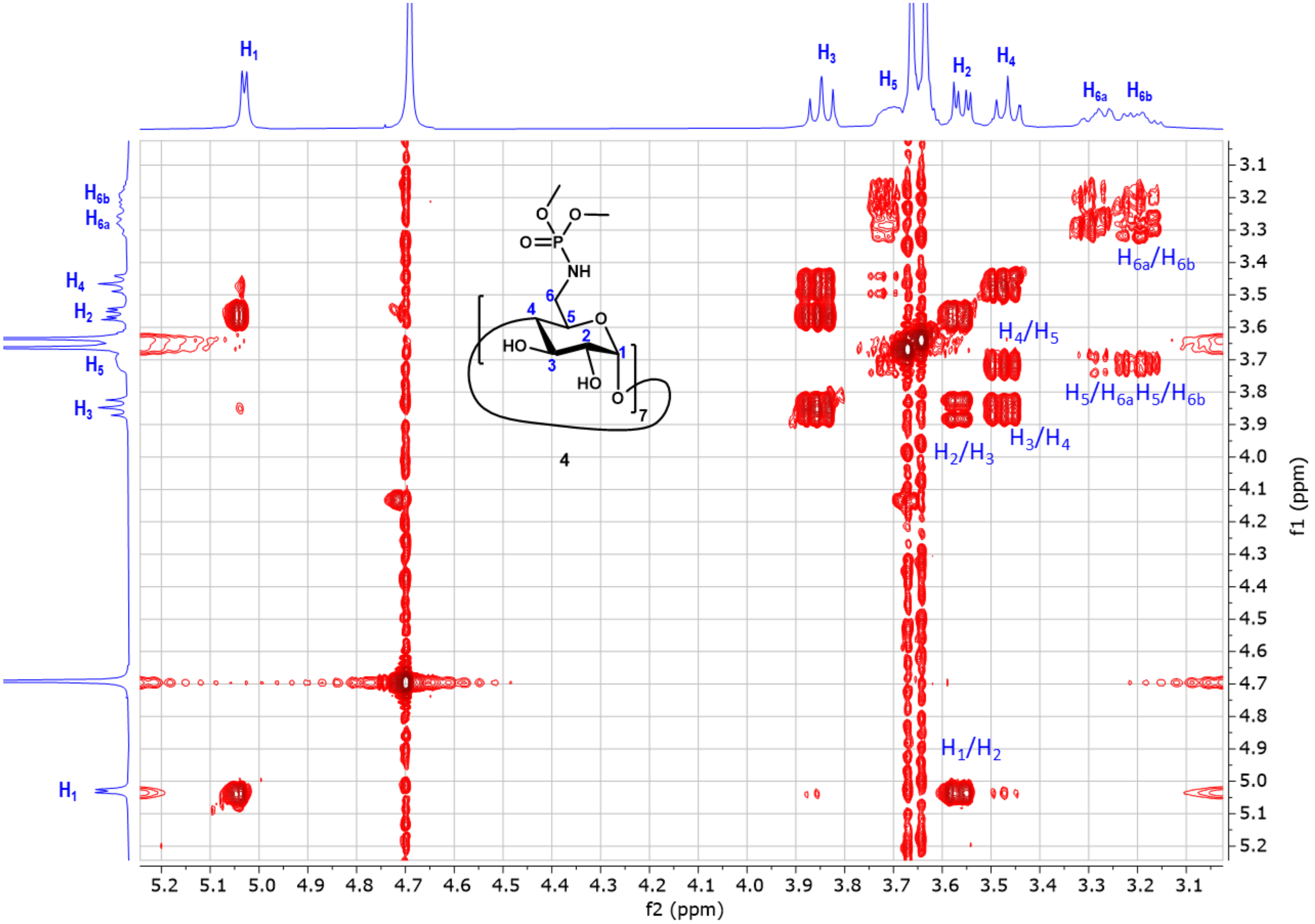

Figure S4: ^{1}H-^{1}H COSY NMR Spectrum of compound **4** in D_2O.

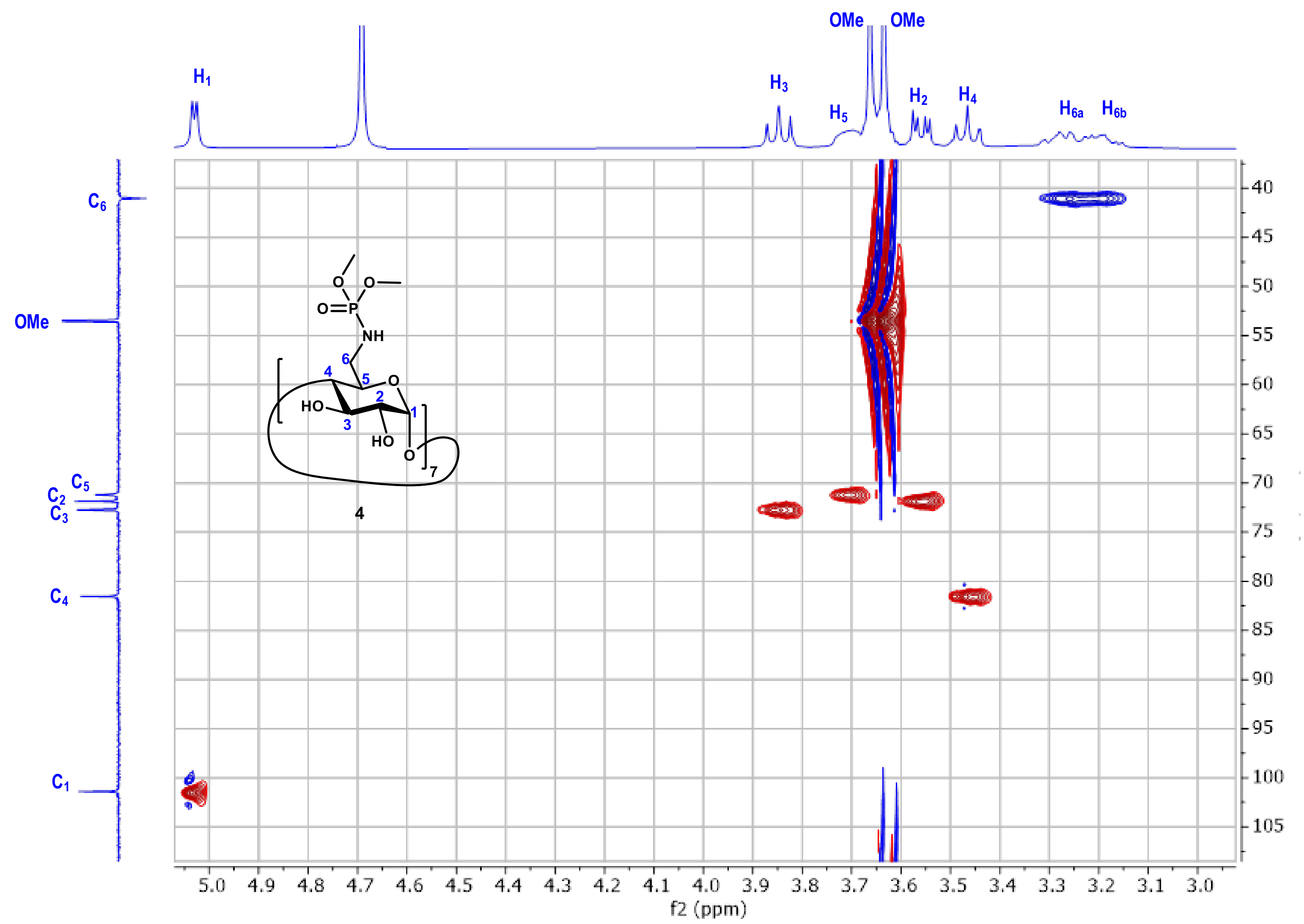

Figure S5: ^{1}H-^{13}C HSQC NMR Spectrum of compound **4** in D_2O.

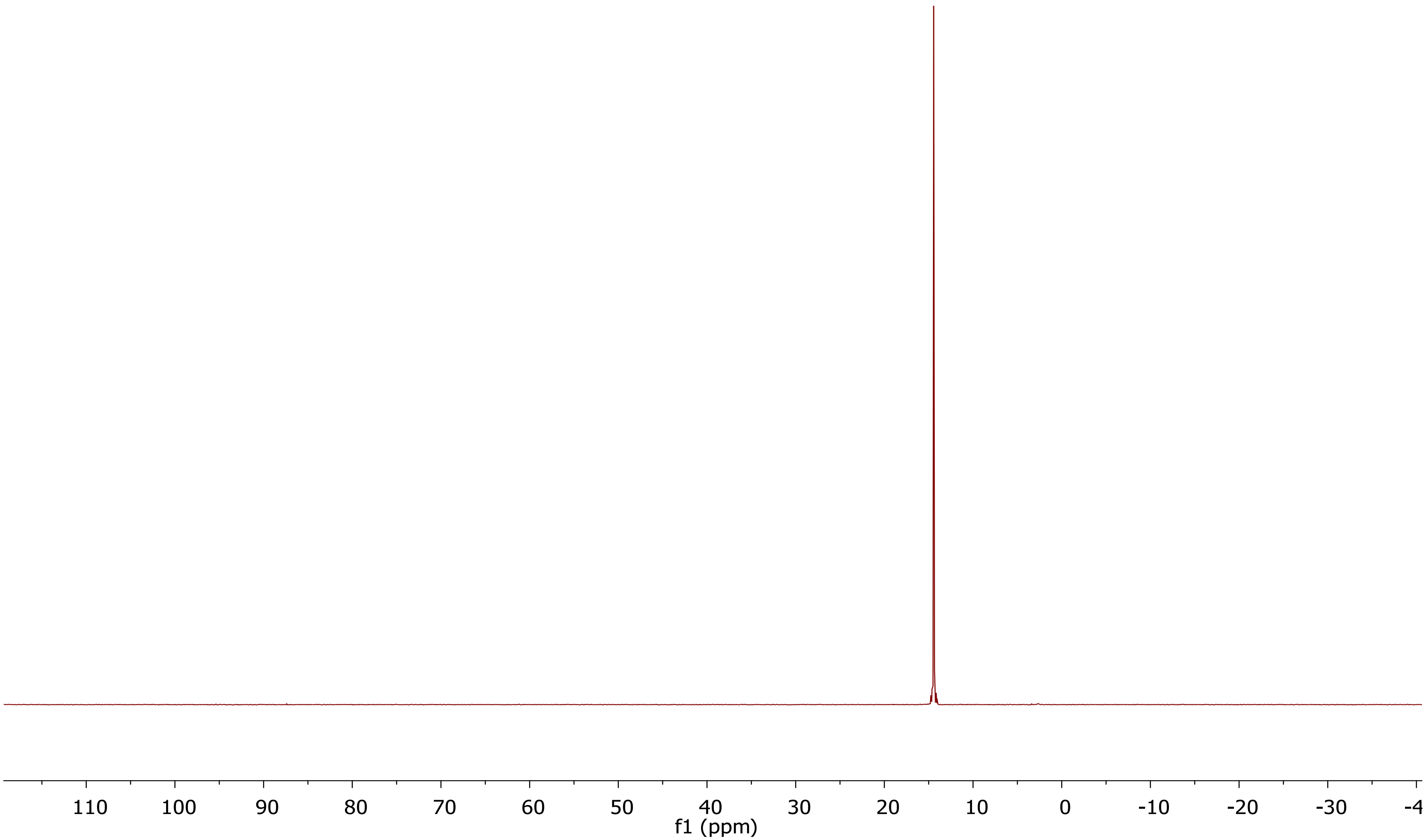

Figure S6: ^{31}P-NMR Spectrum of compound **4** in D_2O.

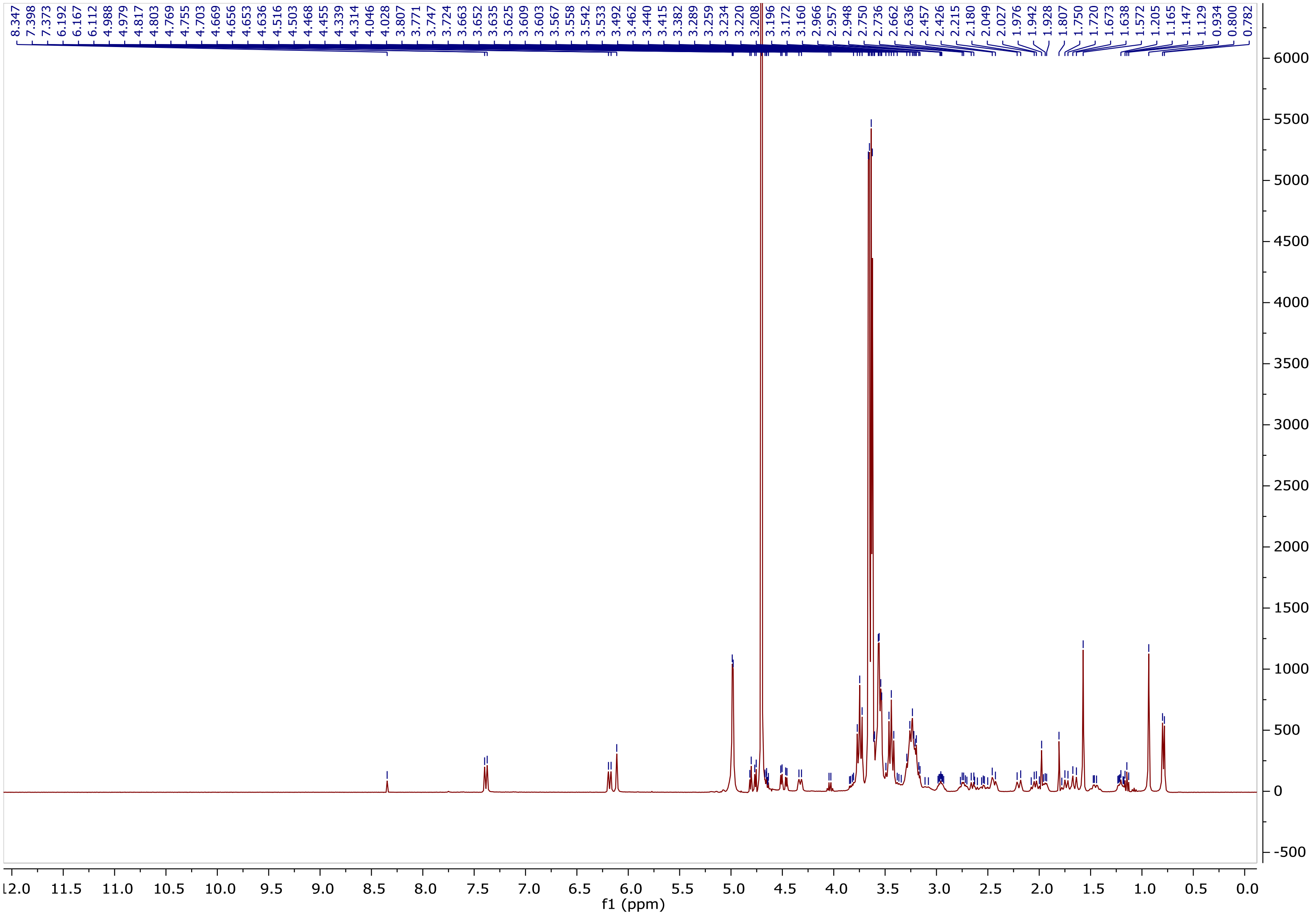

Figure S7: ^{1}H NMR Spectrum of compound **4** + Dexamethasone Sodium Phosphate (Dex-P, **7**) in D_2O.

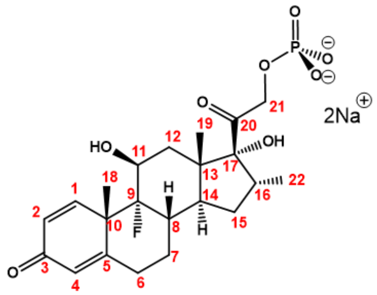

7

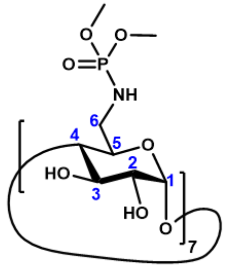

4

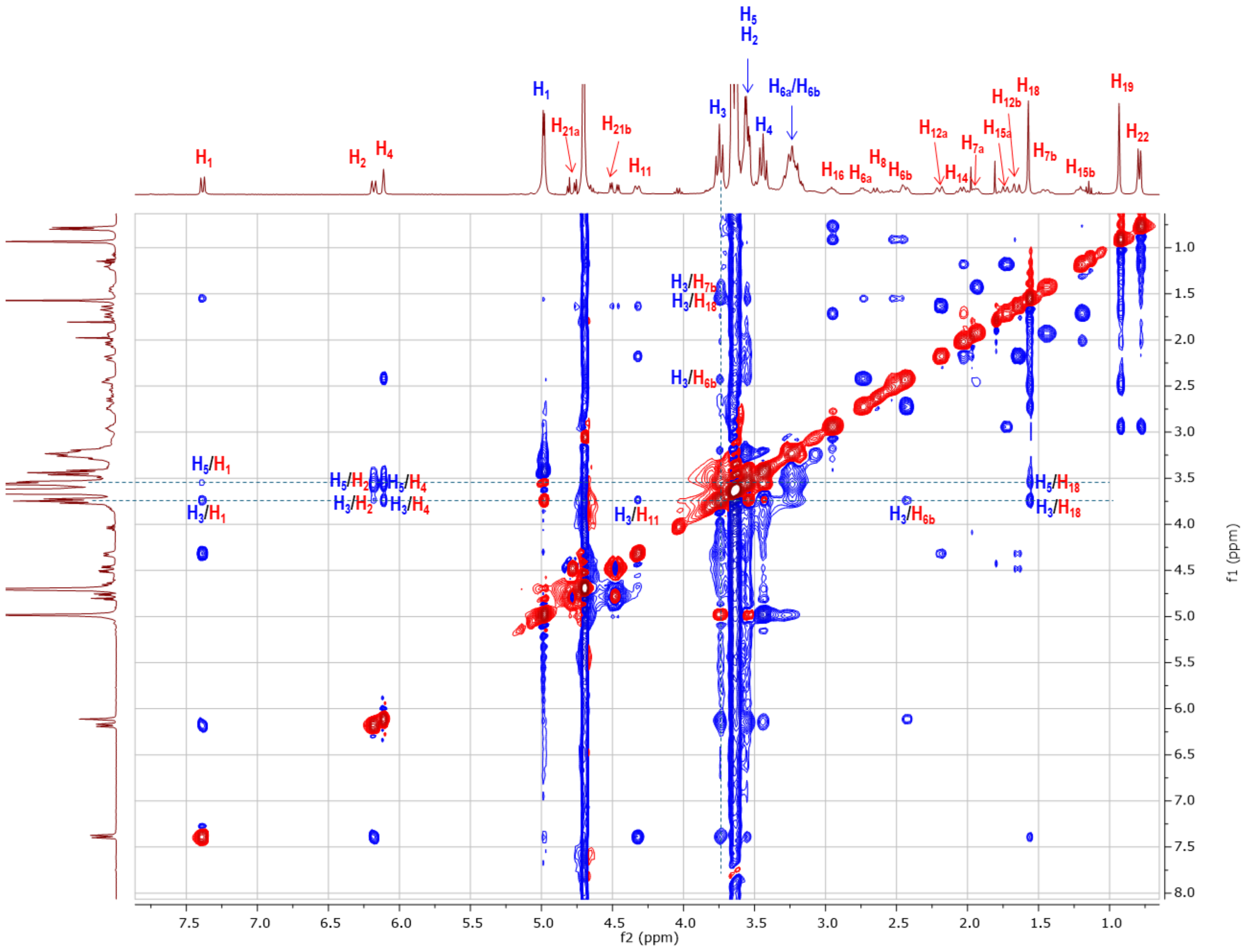

Figure S8: ^{1}H–^{1}H ROESY Spectrum of compound **4** + Dexamethasone Sodium Phosphate (DEX-P, **7**) in D_2O.

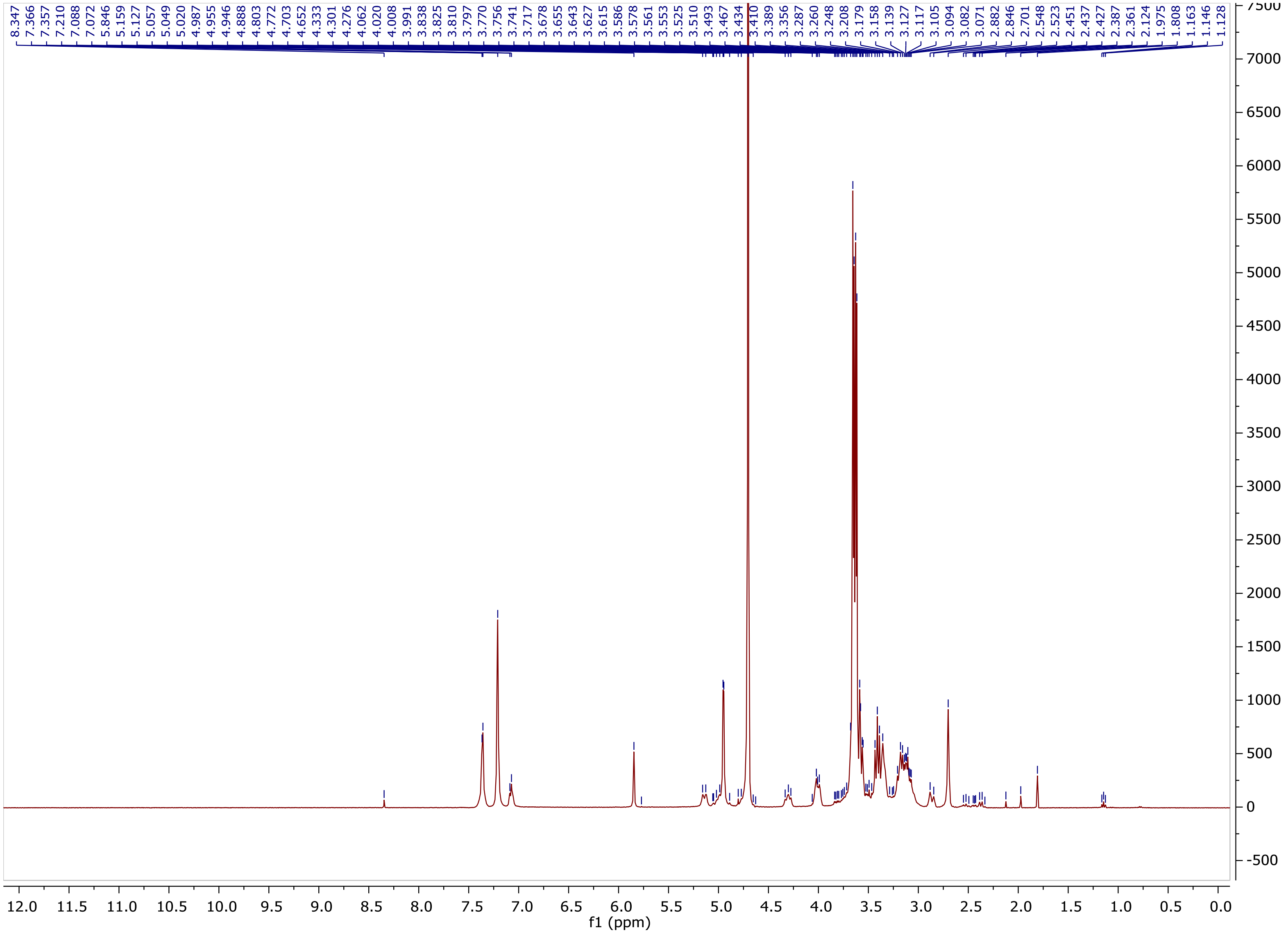

Figure S9: ^{1}H NMR Spectrum of compound **4** + Nefopam HCl (**9**) in D_2O.

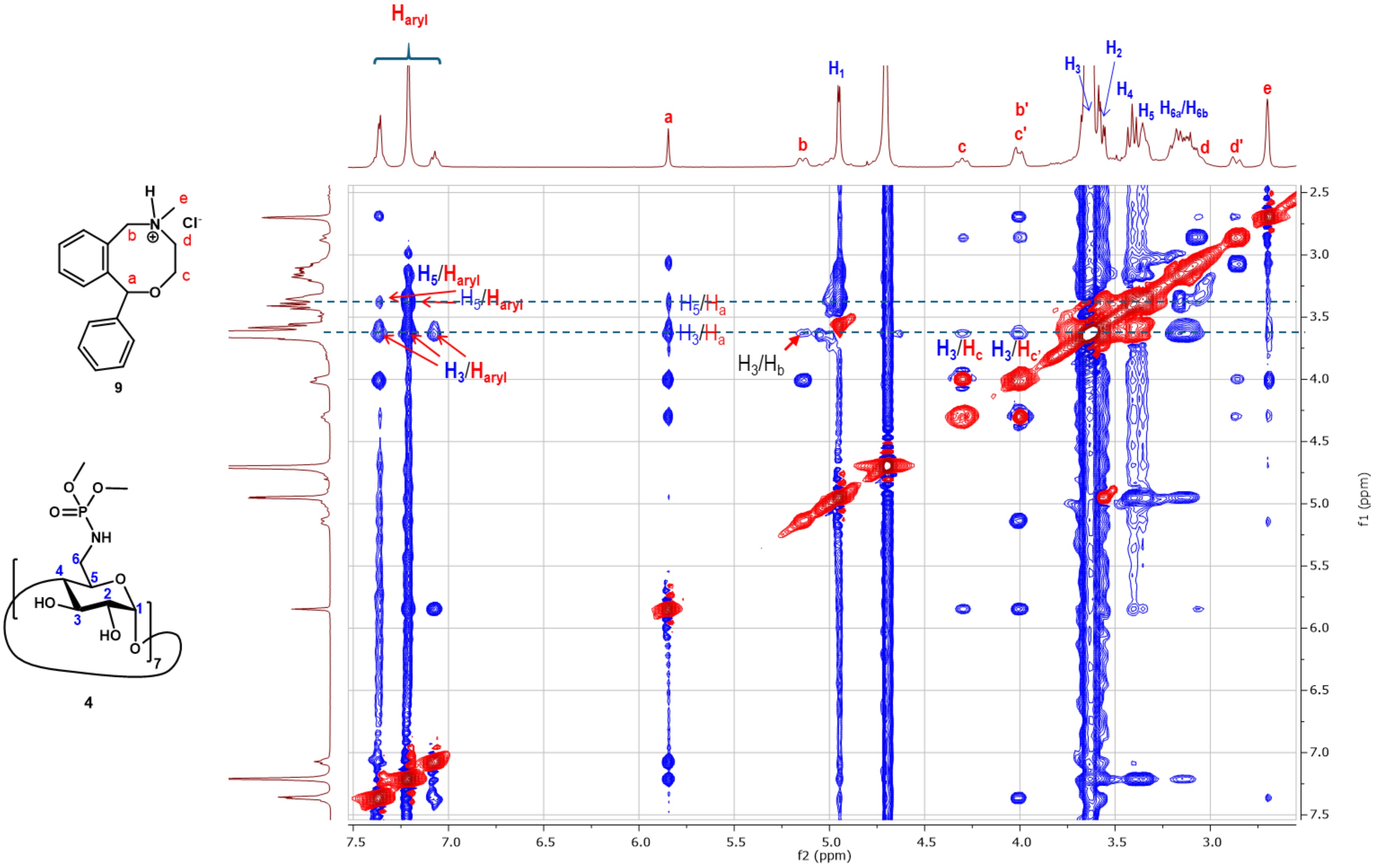

Figure S10: ^{1}H–^{1}H ROESY Spectrum of compound **4** + Nefopam HCl (**9**) in D_2O.

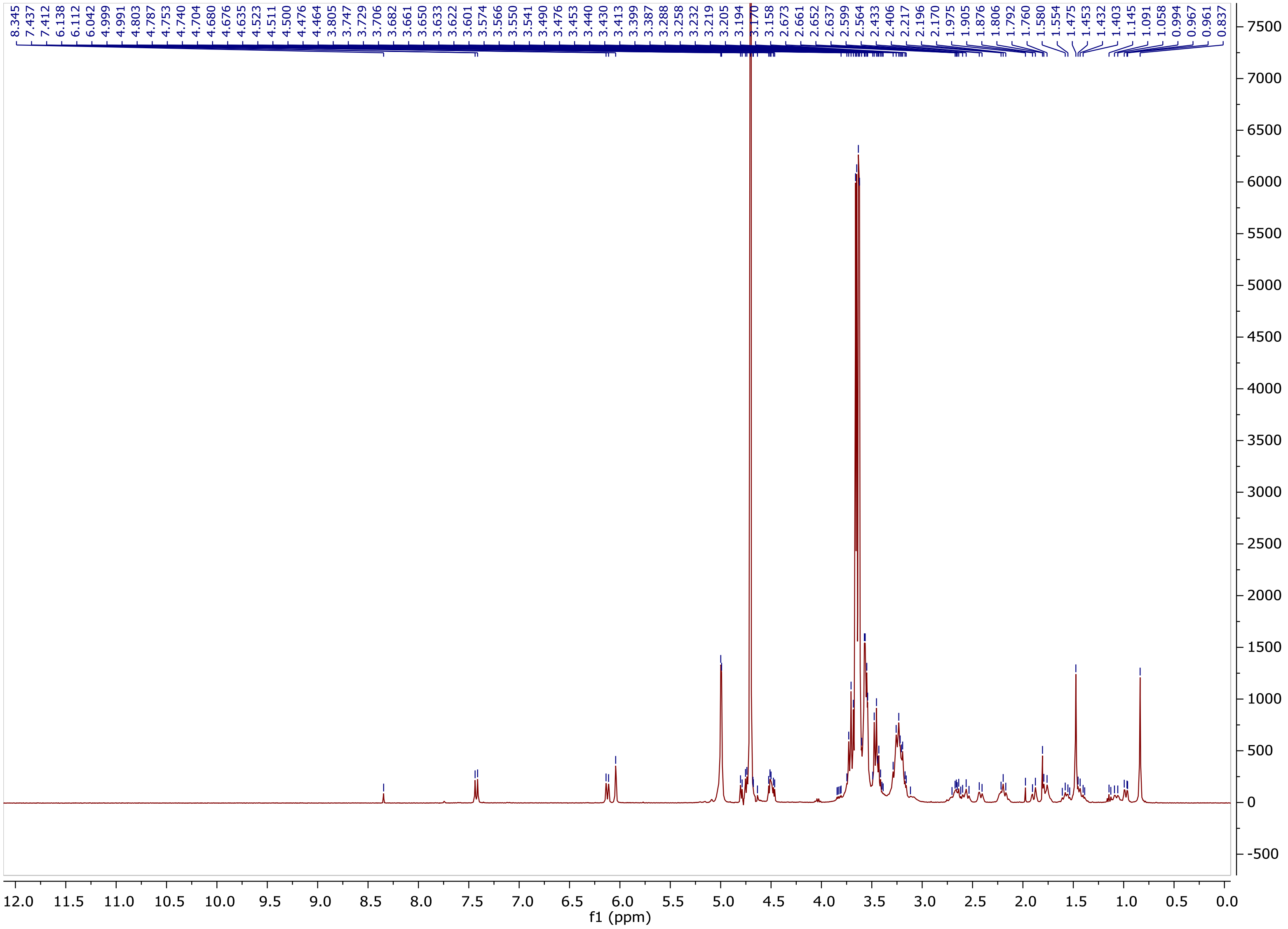

Figure S11: ^{1}H NMR Spectrum of compound **4** + Prednisolone Disodium Phosphate (Prd-P, **8**) in D_2O.

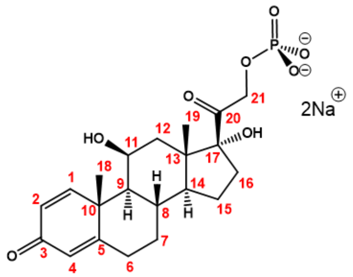

8

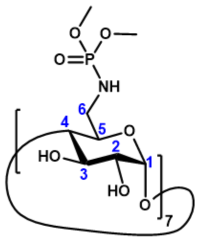

4

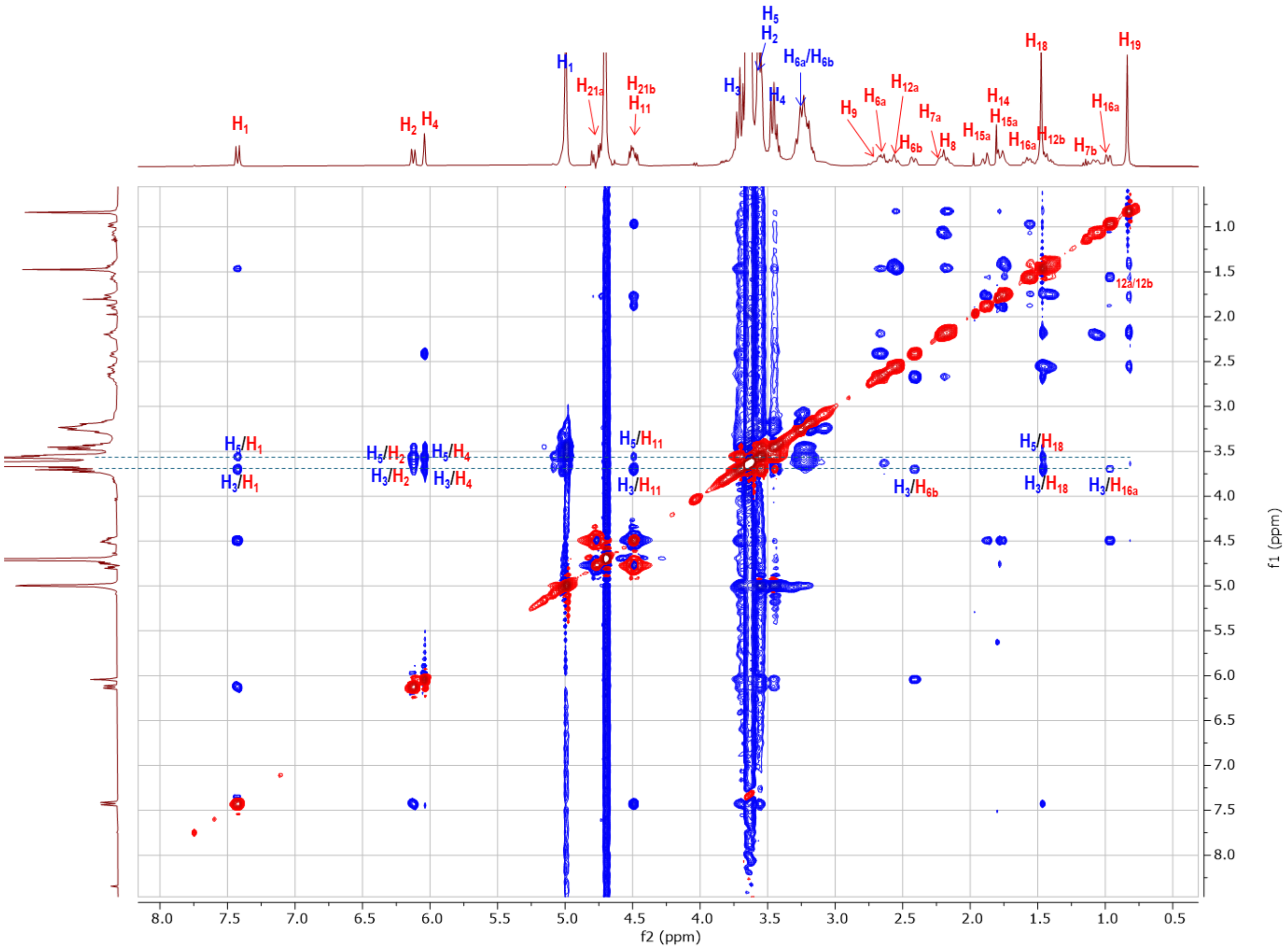

Figure S12: ^1H–^1H ROESY Spectrum of compound **4** + Prednisolone Disodium Phosphate (**8**) in D_2O.

II. Isothermal Titration Calorimetry (ITC)

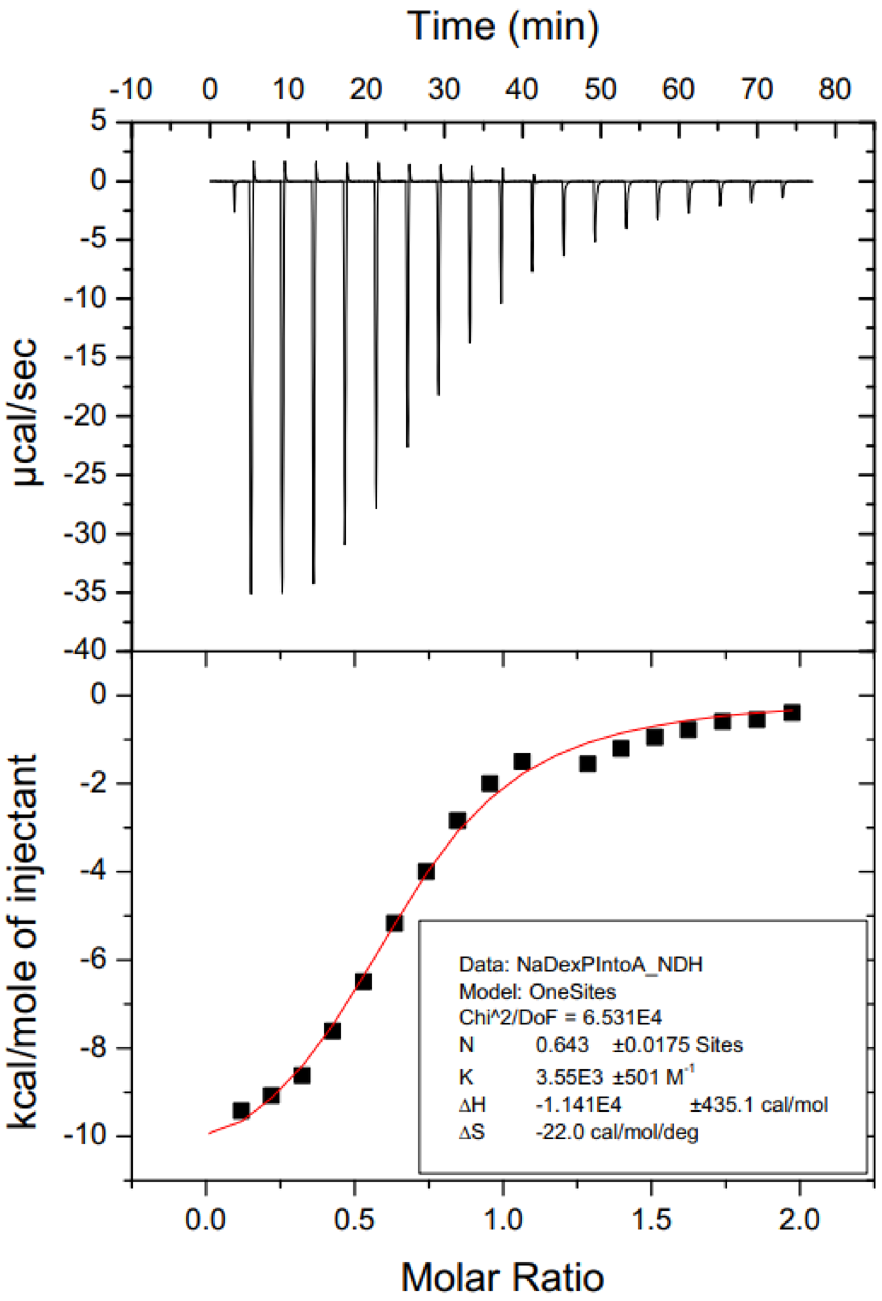

Figure S13: ITC Titration of Dex-P (**7**) into compound **4.**

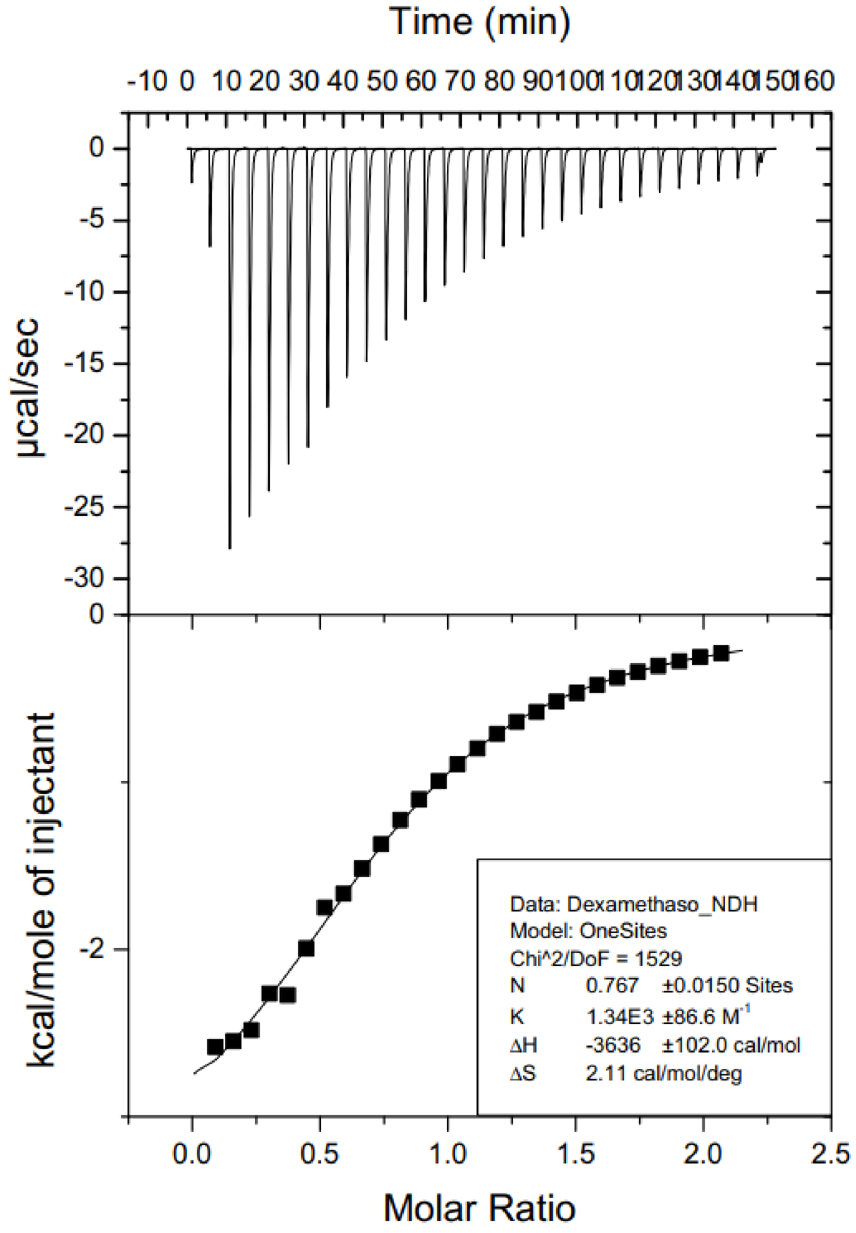

Figure S14: ITC Titration of Dex-P (7) into HPBCD (1).

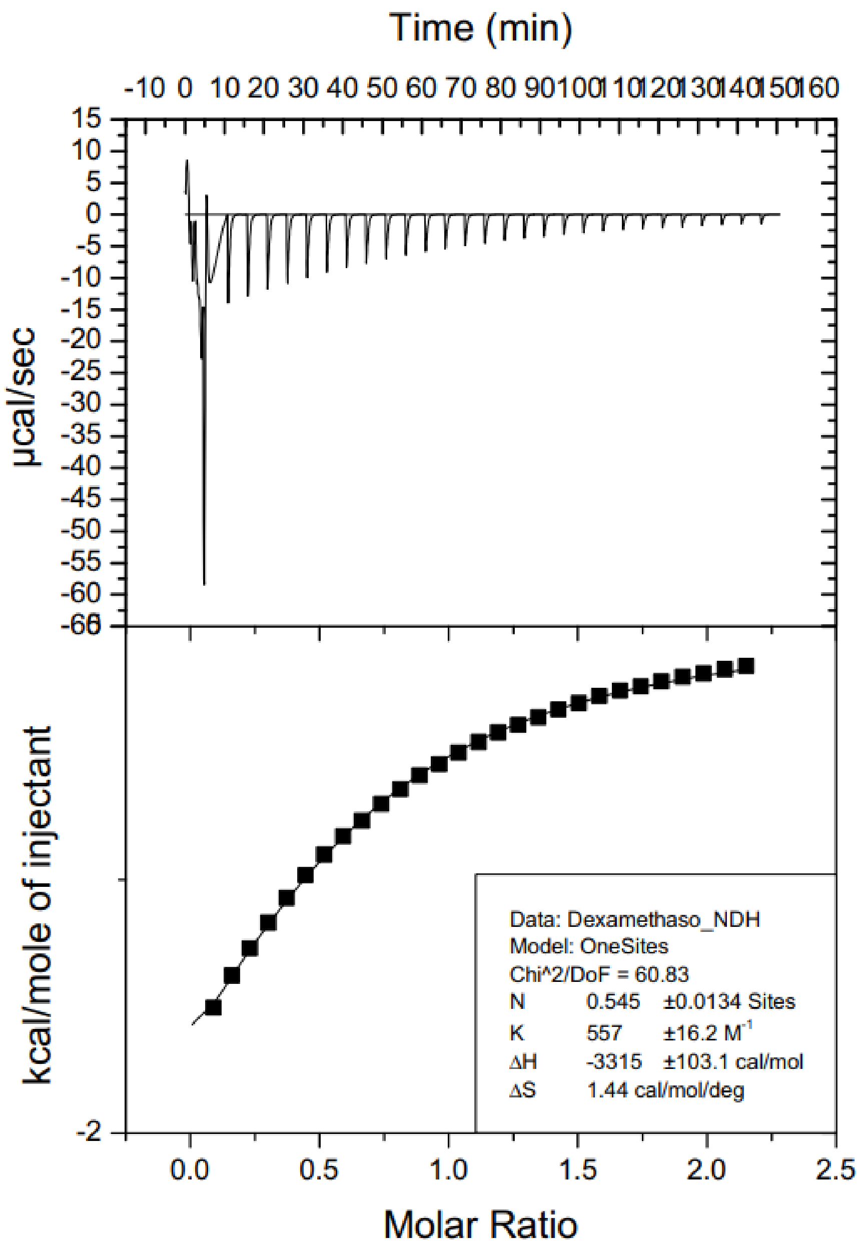

Figure S15: ITC Titration of Dex-P (7) into RMBCD (2).

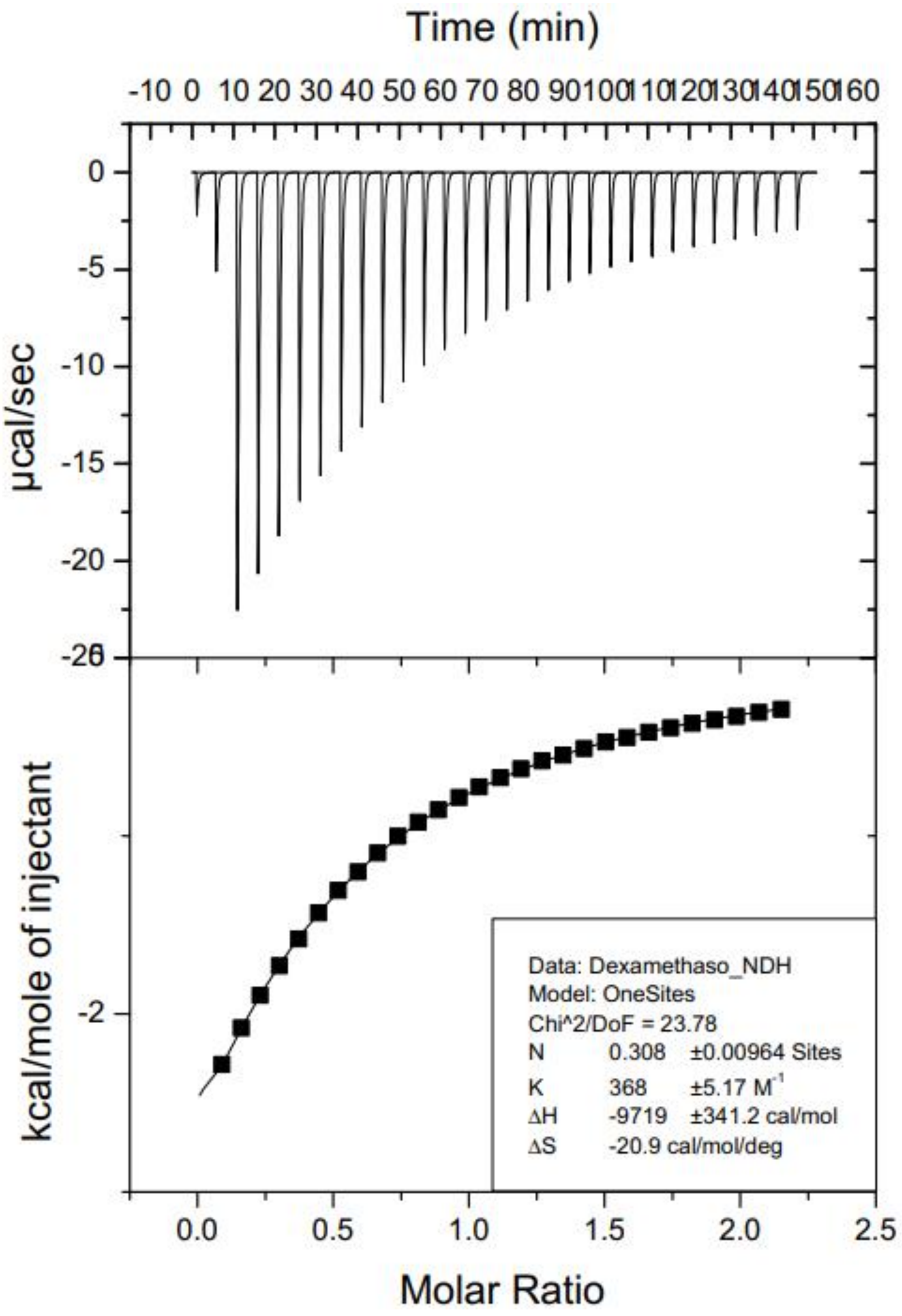

Figure S16: ITC Titration of Dex-P (**7**) into SBEBCD (**3**).

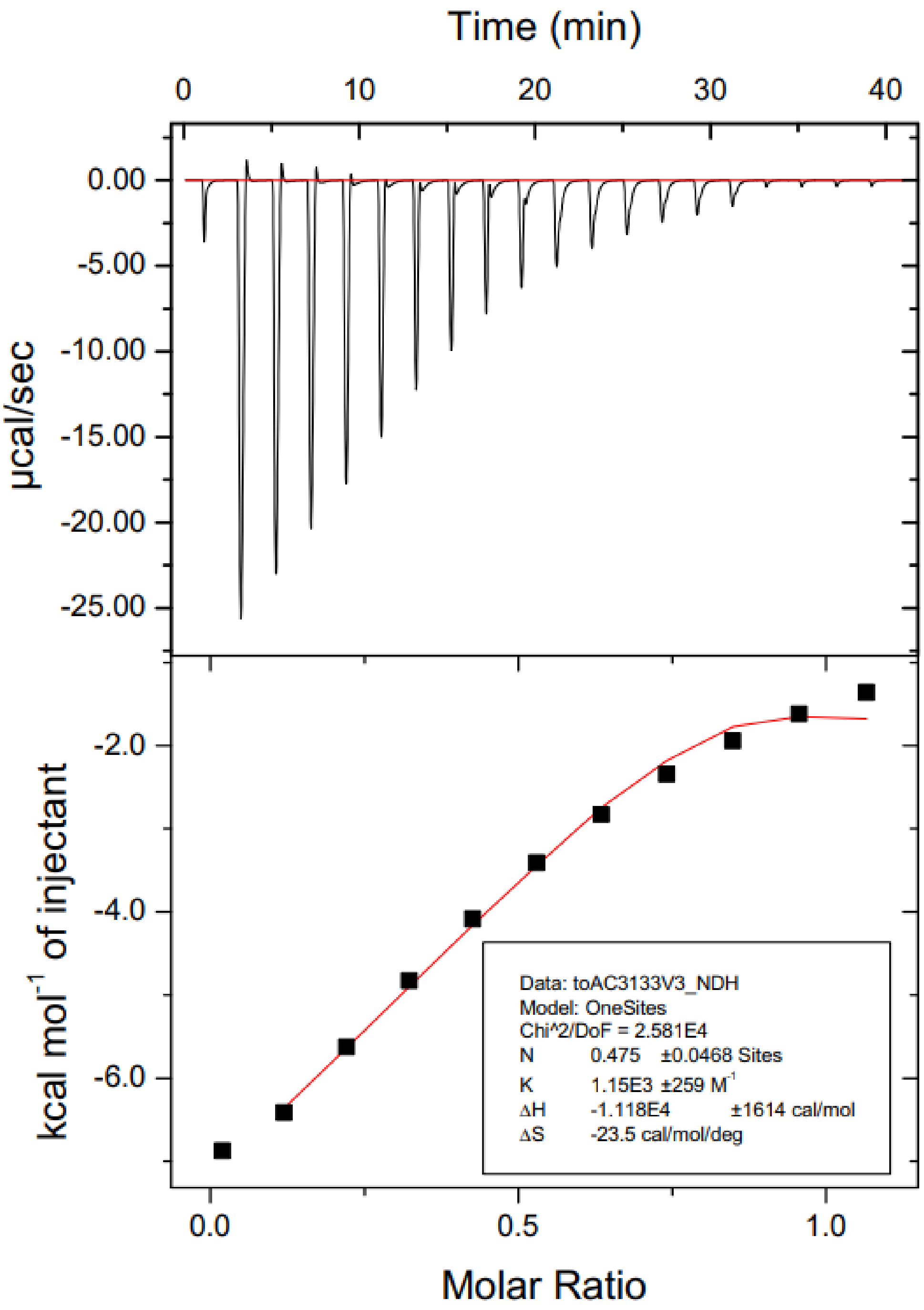

Figure S17: ITC Titration of Prd-P (**8**) into compound **4**.

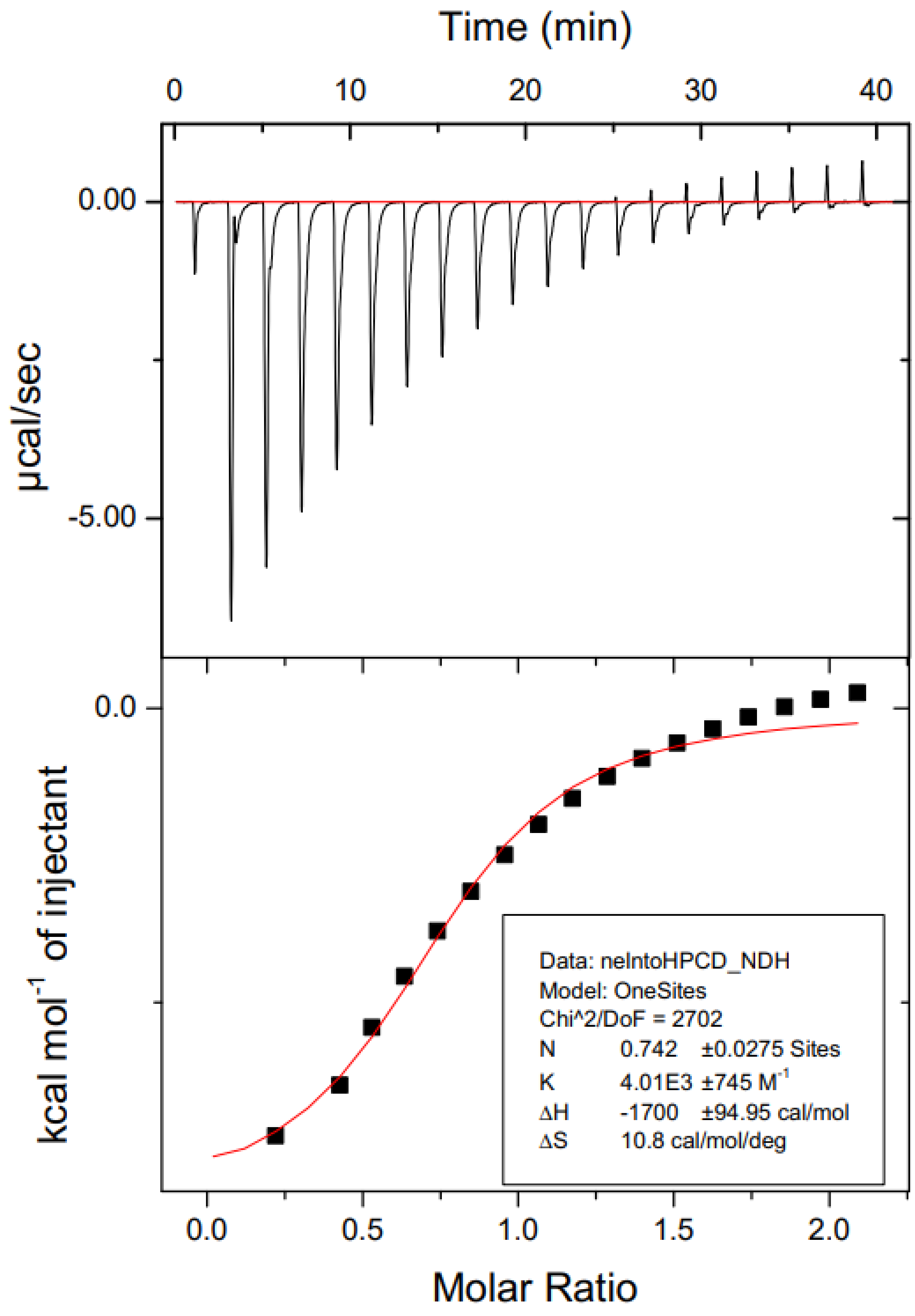

Figure S18: ITC Titration of Prd-P (**8**) into HPBCD (**1**).

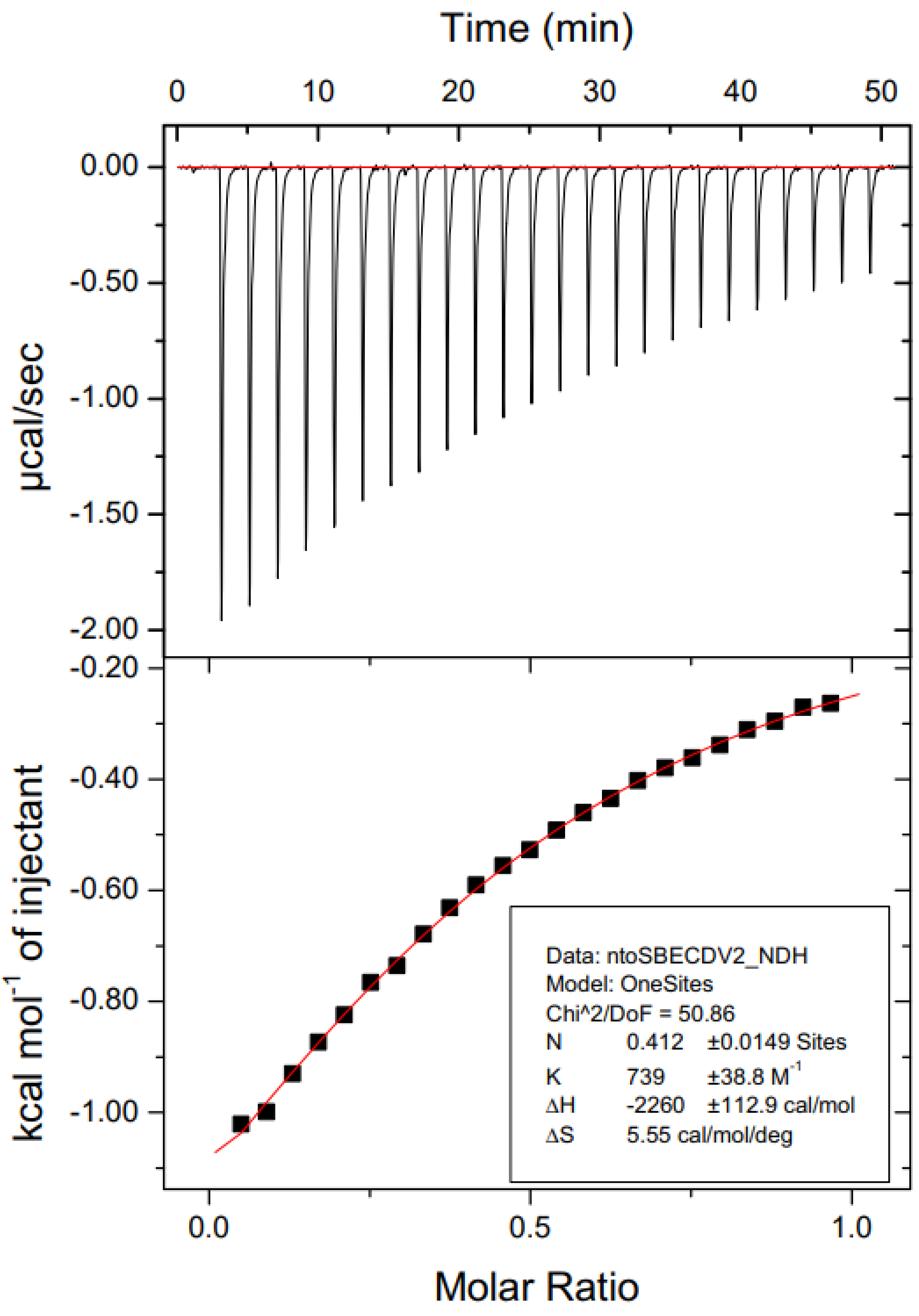

Figure S19: ITC Titration of Prd-P (**8**) into SBEBCD (**3**).

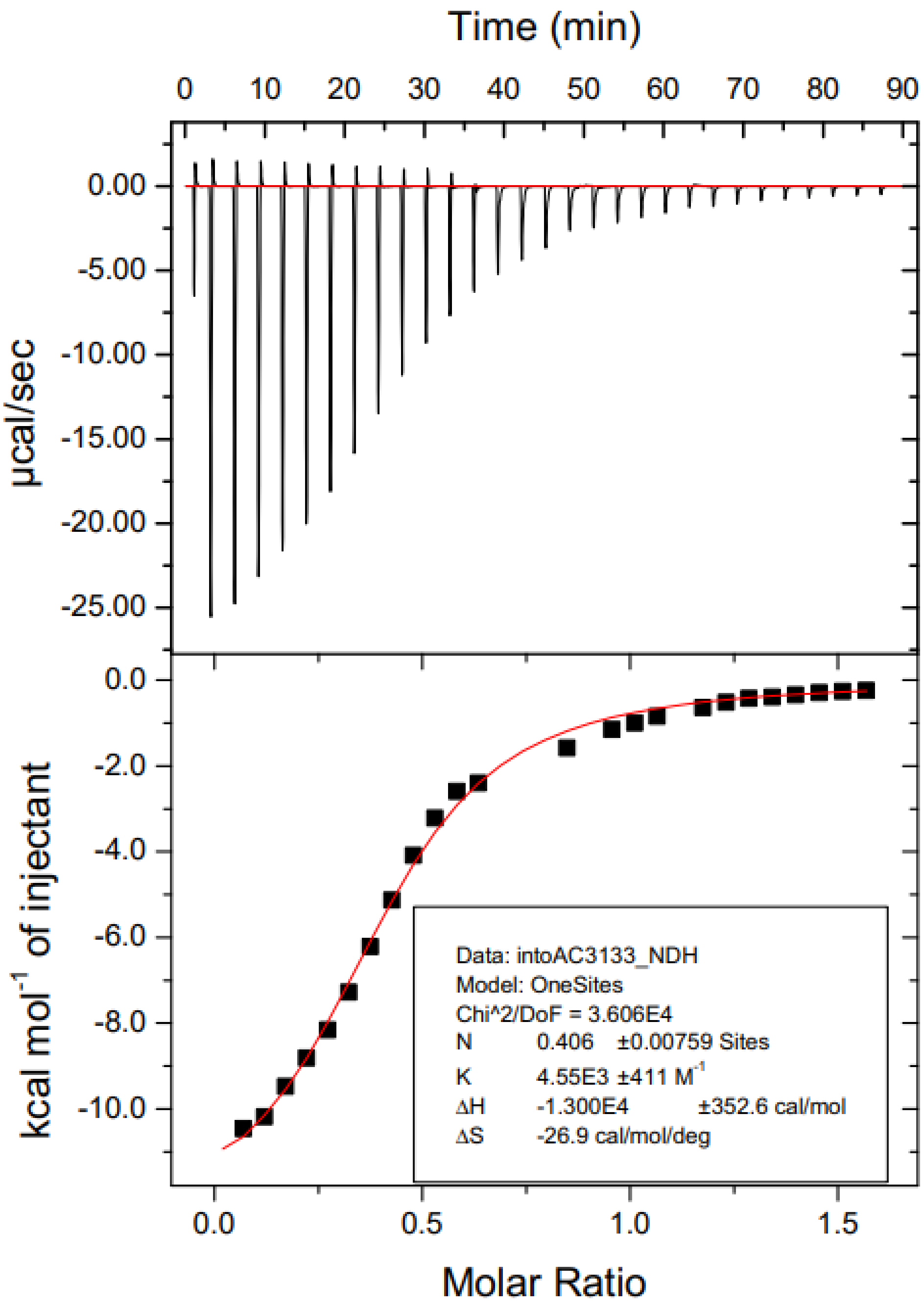

Figure S20: ITC Titration of Nefopam HCl (9) into compound 4 .

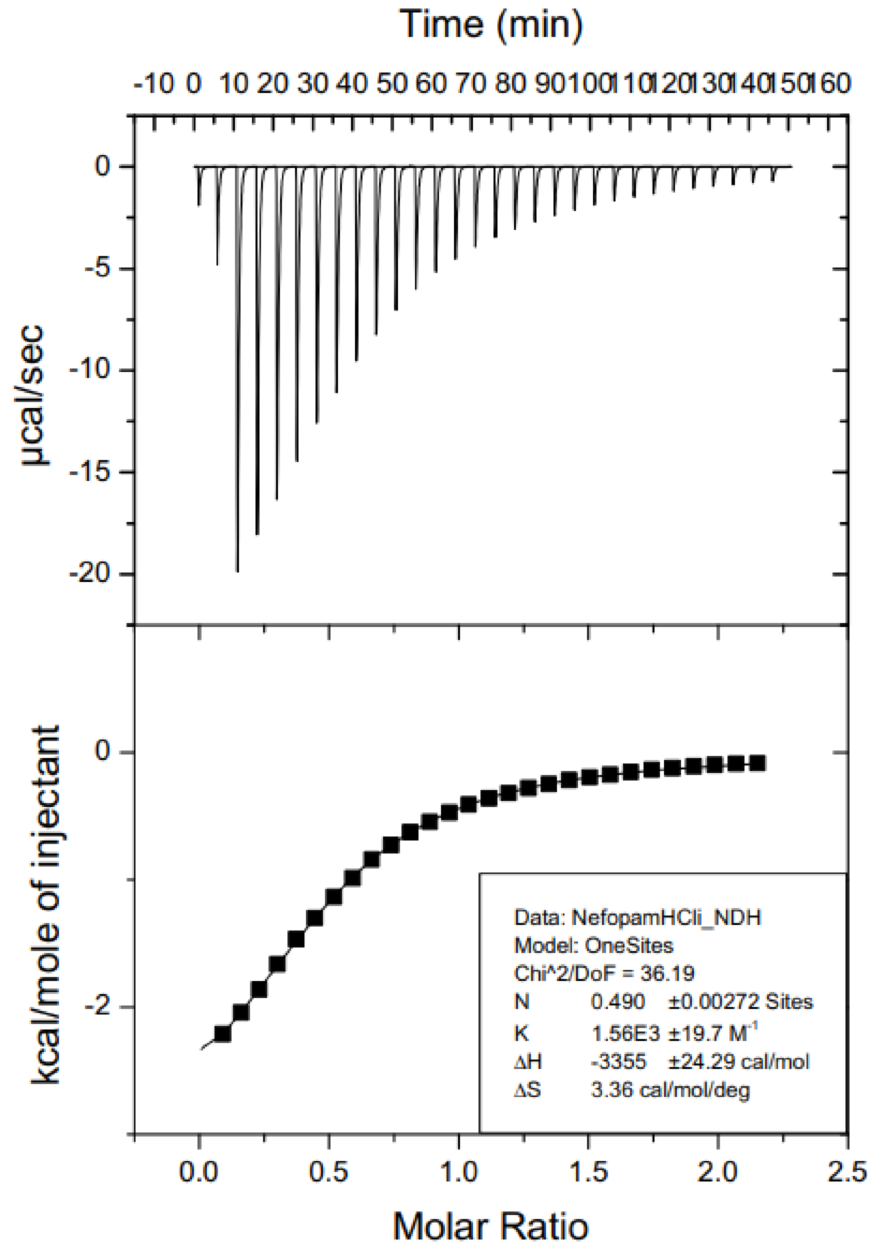

Figure S21: ITC Titration of Nefopam HCl (9) into HPBCD (1).

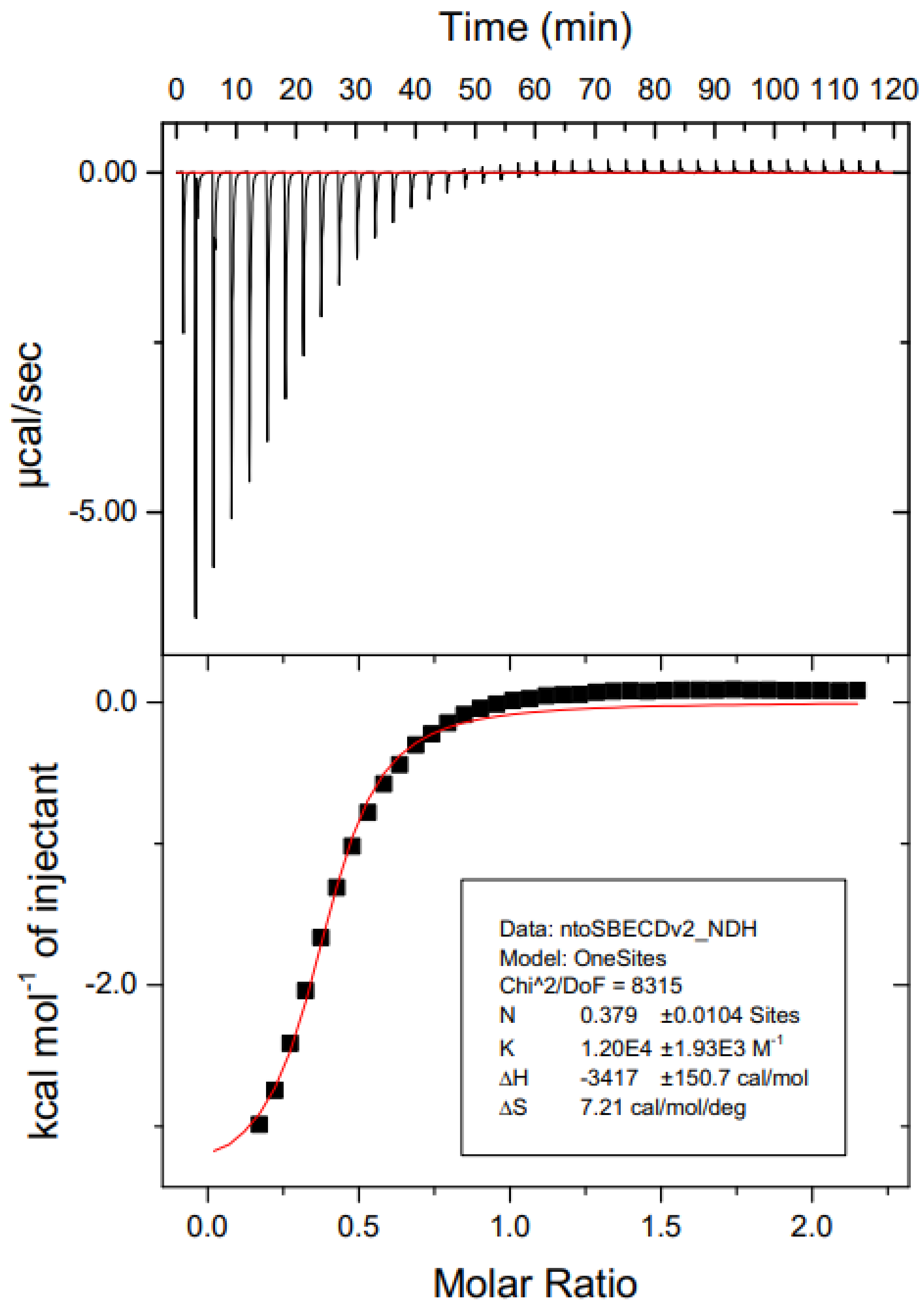

Figure S22: ITC Titration of Nefopam HCl (9) into SBEBCD (3).

HRMS Spectra

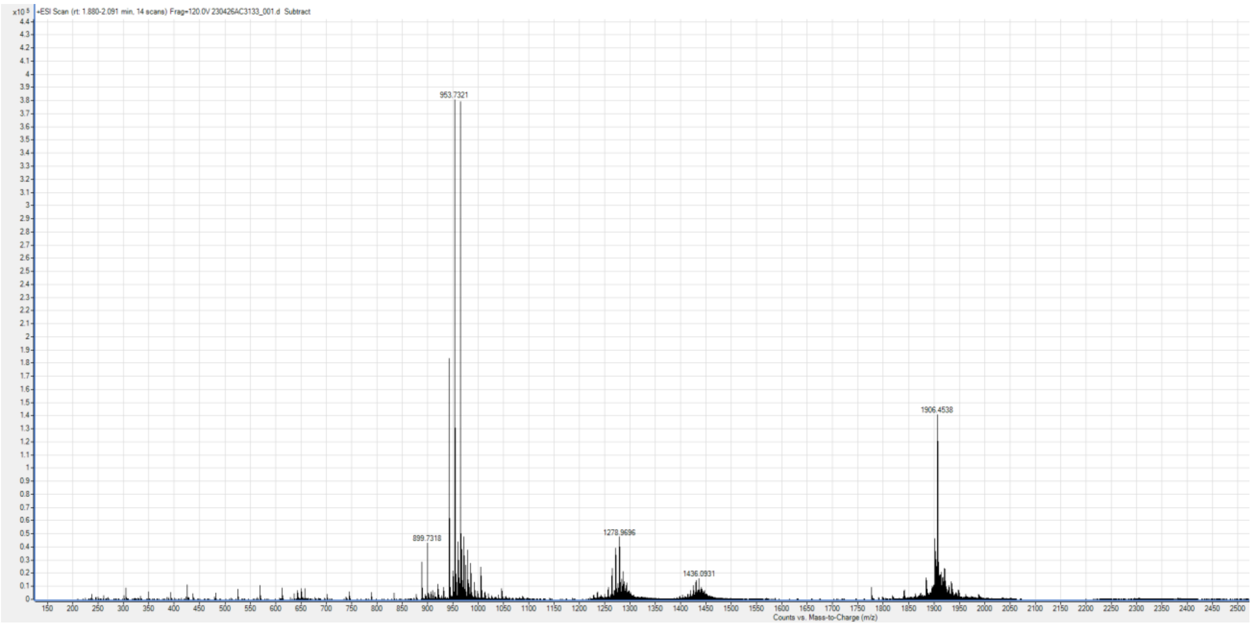

Figure S23: HRMS Spectra of Compound **4**
